# Genome-wide identification of the Fermentome; genes required for successful and timely completion of wine-like fermentation by *Saccharomyces cerevisiae*

**DOI:** 10.1186/1471-2164-15-552

**Published:** 2014-07-03

**Authors:** Michelle E Walker, Trung D Nguyen, Tommaso Liccioli, Frank Schmid, Nicholas Kalatzis, Joanna F Sundstrom, Jennifer M Gardner, Vladimir Jiranek

**Affiliations:** School of Agriculture, Food and Wine, University of Adelaide, Urrbrae, SA 5064 Australia; Wine Innovation Cluster, Adelaide, South Australia

## Abstract

**Background:**

Wine fermentation is a harsh ecological niche to which wine yeast are well adapted. The initial high osmotic pressure and acidity of grape juice is followed by nutrient depletion and increasing concentrations of ethanol as the fermentation progresses. Yeast’s adaptation to these and many other environmental stresses, enables successful completion of high-sugar fermentations. Earlier transcriptomic and growth studies have tentatively identified genes important for high-sugar fermentation. Whilst useful, such studies did not consider extended growth (>5 days) in a temporally dynamic multi-stressor environment such as that found in many industrial fermentation processes. Here, we identify genes whose deletion has minimal or no effect on growth, but results in failure to achieve timely completion of the fermentation of a chemically defined grape juice with 200 g L^−1^ total sugar.

**Results:**

Micro- and laboratory-scale experimental fermentations were conducted to identify 72 clones from ~5,100 homozygous diploid single-gene yeast deletants, which exhibited protracted fermentation in a high-sugar medium. Another 21 clones (related by gene function, but initially eliminated from the screen because of possible growth defects) were also included. Clustering and numerical enrichment of genes annotated to specific Gene Ontology (GO) terms highlighted the vacuole’s role in ion homeostasis and pH regulation, through vacuole acidification.

**Conclusion:**

We have identified 93 genes whose deletion resulted in the duration of fermentation being at least 20% longer than the wild type. An extreme phenotype, ‘stuck’ fermentation, was also observed when *DOA4*, *NPT1*, *PLC1*, *PTK2*, *SIN3*, *SSQ1*, *TPS1*, *TPS2* or *ZAP1* were deleted. These 93 Fermentation Essential Genes (FEG) are required to complete an extended high-sugar (wine-like) fermentation. Their importance is highlighted in our Fermentation Relevant Yeast Genes (FRYG) database, generated from literature and the fermentation-relevant phenotypic characteristics of null mutants described in the *Saccharomyces* Genome Database. The 93-gene set is collectively referred to as the ‘Fermentome’. The fact that 10 genes highlighted in this study have not previously been linked to fermentation-related stresses, supports our experimental rationale. These findings, together with investigations of the genetic diversity of industrial strains, are crucial for understanding the mechanisms behind yeast’s response and adaptation to stresses imposed during high-sugar fermentations.

**Electronic supplementary material:**

The online version of this article (doi:10.1186/1471-2164-15-552) contains supplementary material, which is available to authorized users.

## Background

*Saccharomyces cerevisiae* strains are widely used for production of alcoholic beverages such as wine, beer, sake, as well as bioethanol. The selection of yeast strains for efficient fermentation performance in these industrial processes has typically focused on attributes such as predictable fermentation at the relevant process temperatures, desired fermentation vigour and extent of sugar attenuation with efficient conversion to ethanol. Attributes deemed important for wine fermentation include retention and enhancement of varietal (grape derived) fruit characters and production of desirable flavour and aroma compounds [1]. The numerous subtle differences in fermentation traits between the various industrial yeast strains are reflected in the overall genetic diversity found in this species [2]. Nevertheless, industrial yeast share the ability to grow and ferment in high sugar media, sensing, responding and adapting to the extreme and changing conditions. Such conditions are imposed by anaerobic conditions, high concentrations of sugar and organic acids (resulting in high osmotic pressure), toxins and inhibitors, at times low pH and assimilable nitrogen levels, extended fermentation times and increasing concentration of ethanol or other inhibitors. Our understanding of the cellular mechanisms behind yeast’s adaptation to the temporal exposure to multiple environmental stresses in such fermentations is limited despite extensive studies in the past decade.

Extensive ‘phenomic’ studies have been undertaken with laboratory yeast collections, comprised of individual known single gene deletion mutants (deletants), whereby the phenotype of such deletants is analysed to determine the genes associated with tolerance to a specific condition. Several studies have looked at tolerance or growth sensitivity to singular conditions related to high sugar fermentation, including high osmolarity (specifically sucrose [3] and glucose [4]), anaerobic growth [5], oxidative stress [6], tolerance to high pressure and low temperatures [7], ethanol [8–10] and acetic acid [11]. These studies typically did not require the completion of fermentation and were conducted under aerobic conditions usually for 1–3 days only.

This study is unique in that it sought to identify genes required by yeast to not only grow in but to complete fermentation of a high sugar medium, that is a chemically defined grape juice, wherein multiple stresses would be experienced simultaneously or sequentially. The non-availability of a deletion library in a prototrophic wine yeast background necessitated the use of the laboratory yeast library, derived from S288c [12]. Although these lab yeast strains are reported to cope poorly with high osmotic pressure as found in grape juice [13], we and others have shown that the S288c derived diploid BY4743 and haploids BY4741 (data not shown) and BY4742 [13], are able to complete fermentation in high sugar media [14, 15], provided their auxotrophic requirements are met.

Previous studies have identified genes essential for growth under particular conditions relevant to fermentation, but not the entire fermentation process itself. Sufficient biomass is critical for fermentation performance; if there is too little, fermentation becomes severely protracted or may even fail to complete. Adequate biomass does not however guarantee fermentation completion, especially in high sugar media. Furthermore, whilst transcriptomic experiments highlighted genes whose expression was significantly altered during fermentation, these genes are not necessarily required for fermentation reliability.

This report relates to the effect of gene deletions on yeast’s ability to complete extended high sugar fermentation. Those deletants whose maximum biomass was at least 70% of the parent but which exhibited protracted (or stuck) fermentation were taken to highlight genes fundamental for successful fermentation in high sugar conditions, such as in wine production. The clustering of these into specific groupings i.e. Gene Ontology (GO) terms provides insight into the many cellular mechanisms behind yeast’s adaptation to fermentation stresses.

## Results and discussion

### A micro-fermentation screen to identify genes required for successful fermentation in a high sugar medium

**Figure 1:**
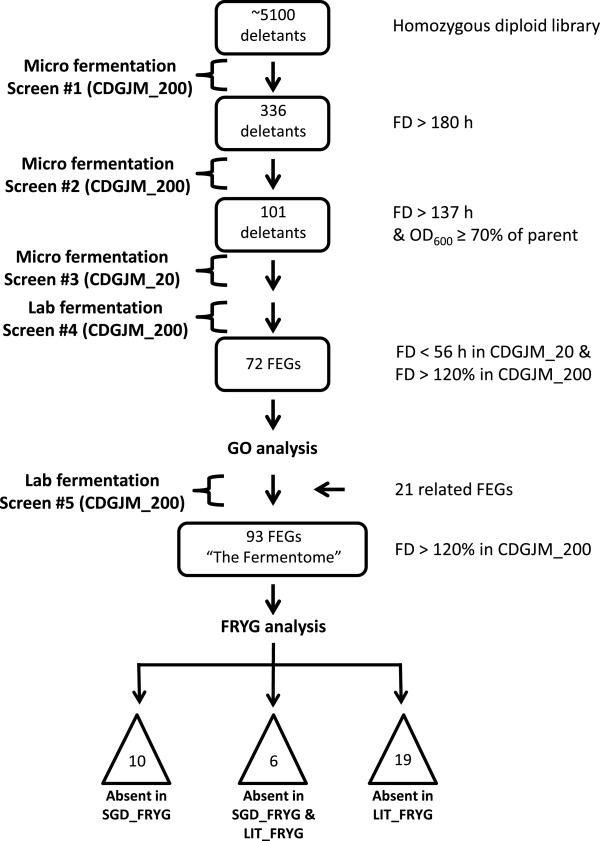
**Outline of fermentation study.** Schematic of the fermentation screen (micro-fermentations; 0.6 mL and laboratory-scale; 100 mL fermentations) and number of gene deletion mutants identified at each stage as having protracted fermentation. Final evaluation using 100 mL fermentations identified 72 candidate genes. *In silico* analysis of fermentation relevant data (SGD_FRYG and LIT_FRYG databases) and GO analysis of genes functionally related to 72 candidate genes. 21 additional gene deletants were identified as resulting in protracted fermentation. Together, the 93 genes are referred to as *F*ermentation *E*ssential *G*enes or the ‘Fermentome’. FD denotes fermentation duration.

In this study (outlined in Figure 1), a high throughput fermentation screen was developed to identify those genes required for high sugar (i.e. akin to wine) fermentation, whereby deletion of the corresponding genes would result in protracted or arrested fermentation. Whereas previous screens have focused on growth assays using standard laboratory media such as YPD supplemented with a stress agent (e.g. ethanol, sugar, oxidizing compounds), our approach was to test the ability of yeast to complete extended fermentation, albeit on a micro-scale. The ability to undergo a wine fermentation requires the yeast to respond and adapt to a dynamic and temporal exposure to multiple stresses in the ‘juice’ environment. Specifically, upon inoculation the cell population must be able to grow to a high density whilst exposed to high osmotic conditions (imposed by the sugars and organic acids in the juice) and the rapid depletion of oxygen and nitrogenous nutrients, sterols and vitamins [16]. With the bulk of fermentation being conducted by cells in stationary phase, long-term adaptation is paramount to the cell’s tolerance to increasing levels of ethanol, as a consequence of sugar uptake and catabolism.

Chemically defined grape juice medium (CDGJM_200) containing 200 g L^−1^ sugar (as equimolar amounts of glucose and fructose) was chosen for the high sugar medium. Whilst fructose and glucose are found in grape must [17] and 200 g L^−1^ of sugars is considerably higher than the 20 g L^−1^ used in laboratory media, this concentration is at the lower end of typical concentrations found in grape must (240–300 g L^−1^ [18]). This compromise was made due to the poorer fermentative ability of laboratory strains in comparison to wine yeast strains [19], in particular the reported sensitivity of S288c derivatives under high osmotic pressure [13]. The assimilable nitrogen content (487 mg N L^−1^) was sufficient for complete fermentation of 200 g L^−1^ of sugar and greater than the minimum requirement by yeast (140 mg N L^−1^ [15]) and also addressed the auxotrophic requirements of the diploid strain, BY4743, in which the yeast homozygous gene deletion collection was generated [12]. At the study’s commencement the lack of availability of a prototrophic yeast deletion collection in either a laboratory yeast [20] or wine yeast genetic background [21] meant that fermentation without the addition of amino acids matched to the auxotrophic requirements of BY4743 could not be investigated. Such an investigation is nonetheless of interest to determine the genetic basis for differences in the nitrogen efficiency of yeast [15].

The initial screen was performed in duplicate 0.6 mL fermentations at 28°C over 7 days in uracil-supplemented CDGJM_200. This medium supported growth of the parental strain BY4743, with all glucose and fructose being consumed within 144 h (data not shown). The individual fermentation performance of each of the ~5100 yeast clones of the homozygous diploid deletion collection was compared with BY4743 (data not shown; Figure 1). The extent of fermentation was defined by enzymatic determination of the residual glucose and fructose content after 180 h and was considered complete when the total residual sugar was <2.5 g L^−1^. The additional 36 h, representing an extra 25% in fermentation duration, was chosen to allow for growth discrepancies due to the potential for minor differences in inoculation rates. 336 gene deletion mutants were identified as having failed to complete fermentation after 180 h (Additional file 1).

In order to eliminate the possibility that incomplete fermentation by these 336 deletants was due to impaired growth, micro-fermentations were repeated with the additional estimation of growth (OD 600 nm) over the duration of the fermentation (Figure 1 and Additional file 1). Residual sugars in the subsequent experiment were analysed at 137 h (BY4743 residual sugar = 2.48 g L^−1^), and only deletants with optical densities (OD_600_) comparable to BY4743 (i.e. > 70%) were chosen for further investigation. In such candidates, the corresponding gene deletion was considered to have no or little effect on growth and to more specifically affect fermentation. As such, 101 deletants were chosen as potential candidates dysfunctional in fermentation under high sugar conditions.

### Evaluation of deletants in laboratory scale (100 mL) fermentation

**Figure 2:**
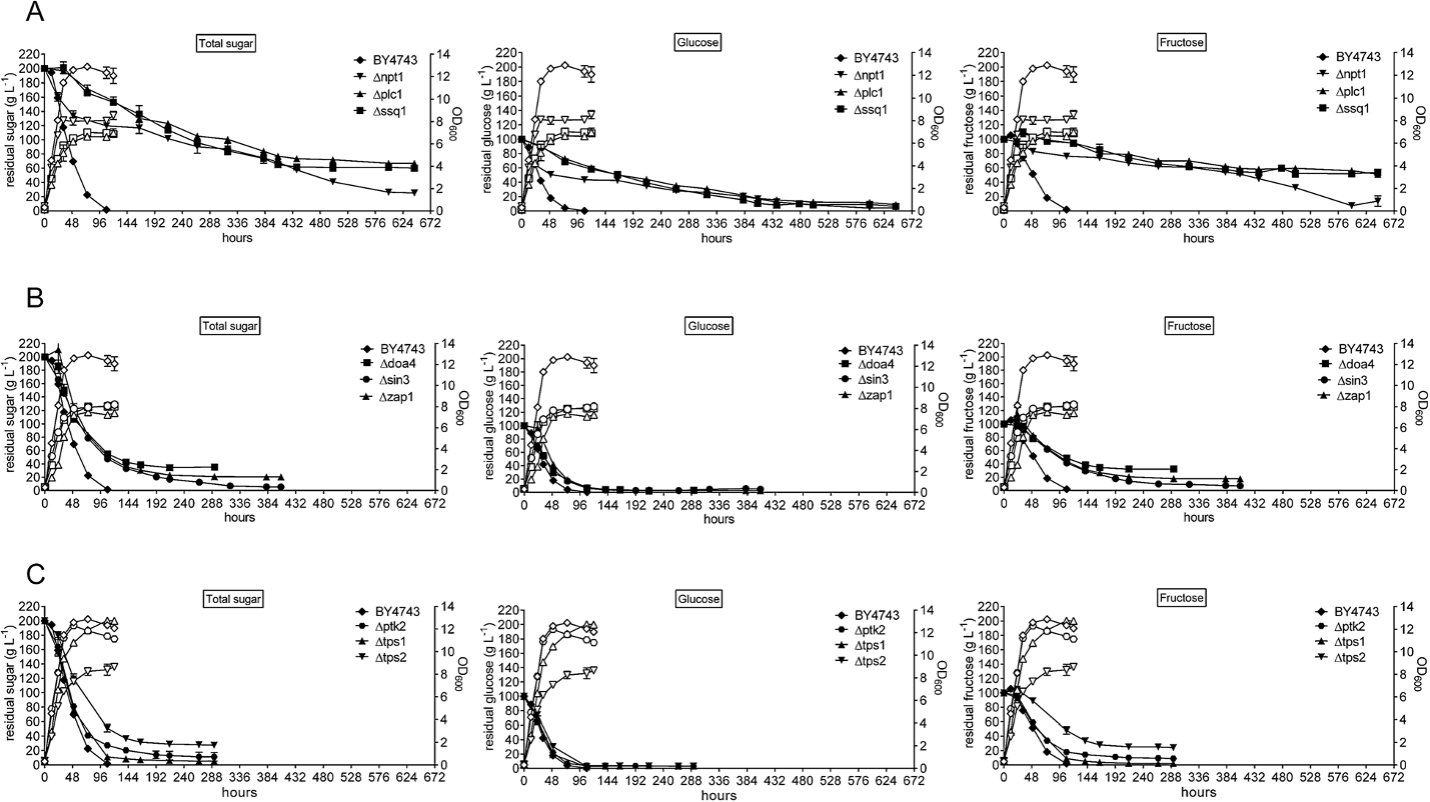
**Comparative fermentations with parent yeast BY4743 and nine stuck mutants in CDGJM_200.** Fermentations (100 mL) were performed in triplicate in CDGJM_200 at 28°C whereby the deletants were compared directly with the parental strain BY4743. Growth was monitored as optical density at 600 nm (open symbols). Sugar consumption was monitored enzymatically and reported as total or individual sugars (solid symbols). The data are arranged in three rows (A-C) of three plots each (left to right). The first plot in each row depicts total residual sugar. Glucose-only and fructose-only information is shown to the right of the corresponding total sugar plot. The nine deletion strains (deletants) which result in arrested or ‘stuck’ fermentation are shown in rows A (Δnpt1, Δplc1, Δssq1), B (Δdoa4, Δsin3, Δzap1) and C (Δptk2, Δtps1, Δtps2).

To test that the fermentation phenotype of the 101 deletants was reproducible in a more controlled environment, the performance of these was compared with the parent strain in larger, 100 mL fermentations (Figure 1). Fermentations conducted at this scale allow greater control over parameters which influence experimental reproducibility and fermentation outcome, such as inoculum preparation, maintenance of anaerobiosis and the inclusion of biological triplicates. The relative fermentation duration (RFD) of deletant versus parent was recorded in the experimental data (Additional file 1). Seventy two deletants had protracted fermentation, taking 20% or longer to complete fermentation than the parent. Eight of these were not only protracted but did not complete fermentation (Figure 2) and the fermentations were deemed ‘stuck’.

The remaining 29 mutants exhibited fermentation durations similar to the parent (14 deletants, RFD = 1.0; 13 deletants, RFD = 1.1; 2 deletants, RFD <1). These findings indicate that although there was a 29% over-estimation of genes affecting fermentation in the second screen (where optical density was measured), 71% of the clones were still identified as affecting fermentation.

The 72 deletants identified as being protracted in fermentation in a high sugar medium (CDGJM_200) were screened as part of the original 336 (Figure 1) in a micro-fermentation of low sugar medium (CDGJM_20) containing 20 g L^−1^ (Additional files 1 and 2). The 72 deletants could be separated into two distinct groups based on two time points at which residual sugar was measured during fermentation: 32 h (fermentation completed by the parent) and 56 h (175% duration). At 32 h, the cut-off of 2.5 g L^−1^ residual sugar was used; 43 deletants were found to have completed fermentation, with 29 deletants having >2.5 g L^−1^ sugar. At 56 hours all the deletants except Δtkl1 had completed fermentation. *TKL1* was retained in the 72 gene dataset based on the delay in fermentation when examined in 100 mL scale using CDGJM_200.

### Use of the FRYG databases to identify genes which modulate fermentation - the ‘Fermentome’

Two FRYG databases (*F*ermentation *R*elevant *Y*east *G*enes) were separately compiled from previously published fermentation relevant studies and the *Saccharomyces* Genome Database (Additional file 3). The databases were complementary; the first (LIT_FRYG) was derived from relevant literature, examining single stress conditions related to fermentation. The second (SGD_FRYG), was compiled from fermentation relevant phenotype terms specifically selected from the more general SGD phenotype terms database, whereby only null mutants (deletants) with a specific phenotypic response, i.e. decreased resistance or increased sensitivity were selected. The 72 gene dataset (representing the remaining candidates following 100 mL fermentations) was compared to the two FRYG databases in order to determine which were previously reported (Figure 1). The SGD_FRYG database was more inclusive with 65 out of the 72 genes being previously reported in related conditions whilst only 55 out of the 72 genes were identified in the LIT_FRYG database.

GO analysis of the identified 72 fermentation essential genes highlighted specific biological processes and more importantly, the other gene members for each GO term, that are likely to be important for fermentation. To ensure that these related genes were not incorrectly excluded as ‘false negatives’ they were re-examined here in a follow up screen. Of the 29 mutants examined, 21 gave protracted fermentation in CDGJM_200.

One of these, (Δtps1) did not finish fermentation and was considered ‘stuck’ (Figure 2 and Additional file 2). The 21 genes were cross-referenced to both FRYG datasets (Additional file 3). The genes absent from the databases are shown in Table 1.

**Table 1:** **Fermentation essential genes shown to be absent in either LIT_FRYG or SGD_FRYG databases**

FEGs absent in LIT_FRYG	FEGs absent in SGD_FRYG	FEGs absent in LIT_FRYG and SGD_FRYG
*DUF1* (*YOL087C*)	*HRK1* (*YOR267C*)	*CCZ1* (*YBR131W*)
*GPA2* (*YER020W*)	*CYK3* (*YDL117W*)	*ATG7* (*YHR171W*)
*NHX1* (*YDR456W*)		*HXK1* (*YFR053C*)
*OPI1* (*YHL020C*)		*PUG1* (*YER185W*)
*PUG1* (*YER185W*)		*RXT3* (*YDL076C*)
*RBL2* (*YOR265W*)		*YFL012W*
*RTT103* (*YDR289C*)		*CIS1* (*YDR022C*)
*TOM1* (*YDR457W*)		*SNX4* (*YJL036W*)
*VAC8* (*YEL013W*)		
*YLL007C*		
*ZAP1* (*YJL056C*)		
*GPR1* (*YDL035C*)		
*OPI1* (*YHL020C*)		

The 93 FEG dataset identified after the fermentation screen and GO analysis depicted in Figure 1, was compared with the LIT_FRYG and SGD_FRYG databases (see Additional file 3 to determine which genes were absent and thus unique to this study).

In summary, 93 genes were identified as being required for fermentation, whereby deletion of the gene resulted in the yeast undergoing protracted fermentation in CDGJM_200 (Additional file 2). Together, the 93 genes, are referred to as the FEG dataset (for Fermentation Essential Genes) and comprise the laboratory yeast ‘fermentome’; genes that are essential for the timely completion of fermentation. The ‘fermentome’ is taken to represent the genes/processes required for yeast to sense and respond to the multiple stresses of the juice environment. These cellular processes enable yeast to grow in grape juice and complete fermentation over the extended period of 4–12 days typical of a wine fermentation. The experimental design however did not allow for the identification of genes whose deletion shortened fermentation, as no time point was taken prior to the parent finishing. Independent studies have identified genes which affect carbon and nitrogen flux and thus positively modulate fermentation, although the mechanisms remain unclear. For example the genes *NGR1* and *GID7*, upon disruption, resulted in enhanced catabolism of sugar in a wine and laboratory strain during growth in CDGJM_200 with limiting nitrogen [22]. Also, deletion of *PDA1*, encoding the pyruvate dehydrogenase Eα subunit, was shown to alter carbon flux during anaerobic fermentation, resulting in shortened fermentation, after an extended lag phase (12 h), which the authors suggested was an acclimatisation to the fermentation conditions [23].

The studies chosen to build the LIT_FRYG dataset included those examining growth in response to a single stress relevant to wine fermentation [3–11, 24–26] as well as a transcriptomic study of the wine strain Vin 13 during fermentation of a Riesling juice [27]. Our dataset represents only a small percentage of the genes identified in the above studies, as shown in LIT_FRYG (Additional file 3), relating to growth sensitivity as a measure of stress tolerance. Not surprisingly, the largest representations relate to hypertonic conditions (21.4%) [24], high glucose (17%) [4], ethanol (8.3% [9], 18.4% [9], 14.9% [10]), anaerobic growth (13.6% [26], 8.3% [5]) and sucrose (9.9%) [3] – see Additional file 3. The importance of experimental design is highlighted by the fact that our study contained only 1.79% of genes described as Fermentation Stress Response (FSR) genes [27]. Although Marks and co-workers [27] looked at gene expression in response to increasing ethanol concentration during fermentation, small changes in transcript levels of FSR may not eventuate in altered fermentation kinetics or outcome - a more likely observation with complete deletion as per our study. A similar conclusion was drawn from data relating to growth rate and stress tolerance [28], whereby the authors examined the yeast deletion collection in continuous culture.

Eighteen of the 93 genes, which resulted in protracted fermentation when deleted, were annotated to vacuole function, specifically the vacuolar H^+^ ATPase complex. Within this dataset, 6 genes were identified by relationship rather than in the original fermentation screen (72 gene dataset) due to clonal errors within the library used in the study. New clones were sourced (Invitrogen) and verified by sequencing prior to analysis (100 mL scale). The occurrence of 10 of these genes in the freeze thaw stress dataset (*VMA1*, *VMA2*, *VMA3*, *VMA4*, *VMA7*, *VMA8*, *VMA11*, *VMA13*, *VPH1* and *VPH2*) [29] indicates that cellular mechanisms required for tolerance to freeze-thaw stress [29] may also be needed for successful fermentation. By comparison of our results to previous studies (see Additional file 3), we propose that the role of the vacuole, specifically maintenance of vacuolar acidification (discussed later) is a key mechanism of cellular response and adaptation during the transitory and dynamic stress conditions imposed by acidic pH, high osmolarity (sugar, organic acids), increasing ethanol content and elevated temperatures during fermentation.

**Figure 3:**
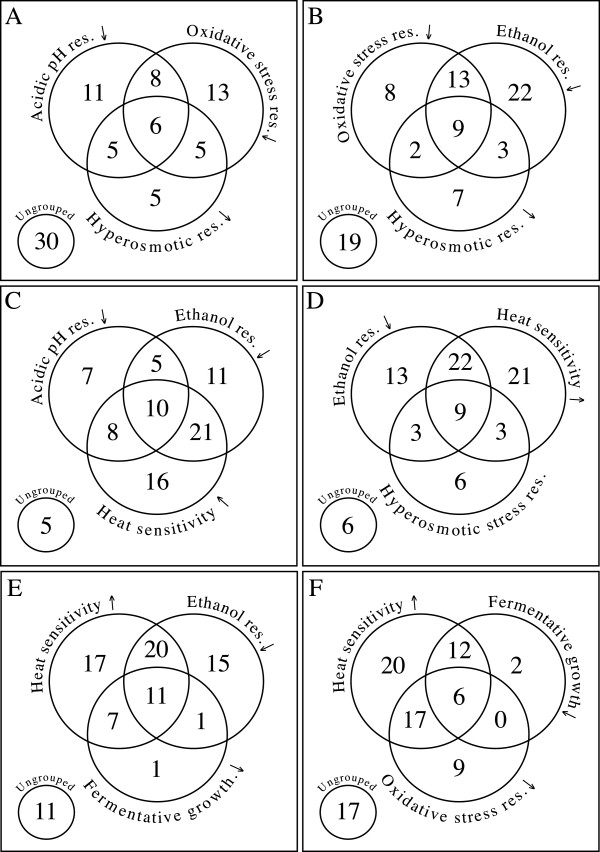
**Comparison of fermentation essential genes with the SGD_FRYG database: phenotypic response to specific stress conditions.** Ninety three fermentation essential genes (FEG) were identified in this study. Ten of the genes have not been previously associated with fermentation related phenotypes and are unique to this fermentation study. The Venn diagrams therefore depict the number of genes within the 83 FEG dataset which upon deletion, leads to either increased sensitivity or decreased resistance to conditions relevant to fermentation, such as anaerobic growth, ethanol toxicity, osmotic and oxidative stress and temperature extremes.

The association between cross-tolerance to multiple stresses and fermentation outcome, as well as fermentative growth and stress tolerance was examined. Genes common to the FEG dataset and selected datasets from the SGD_FRYG database can be highlighted (Figure 3A-F). Here only 83 fermentation essential genes are compared as the additional 10 (of the 93) unique to our study are necessarily excluded. Of particular interest was the large number of genes, when deleted, were associated with decreased resistance to multiple stresses: ethanol, acidic and hyper-osmotic conditions and heat (for example *DOA4* (*YDR069C*), *PLC1* (*YPL268W*), *TPS2* (*YDR074W*), and *SSQ1* (*YLR369W*) as shown in Additional file 3).

Yeast deletants encoding the vacuolar H^+^ ATPase (vma mutants) were often associated with cross tolerance towards several stress conditions but not necessarily fermentative growth. Surprisingly, none of the 83 gene deletions were associated with decreased utilisation of nitrogen and fermentative metabolism (Additional file 3).

### Classification of genes into specific gene ontology categories

Using GO toolbox [30] and GO module [31] the Fermentation Essential genes (FEG) were classified into distinct groups according to GO terms, based on their role in a biological process, function within the cell and cellular localisation (Additional file 4). The frequency of occurrence within the genome of genes annotated to these GO terms was compared to the frequency of occurrence of genes within the FEG dataset annotated to these same terms. An increased frequency of occurrence (an enrichment) of genes in the FEG dataset annotated to a specific GO term implies the importance of that GO cluster to fermentation completion.

**Figure 4:**
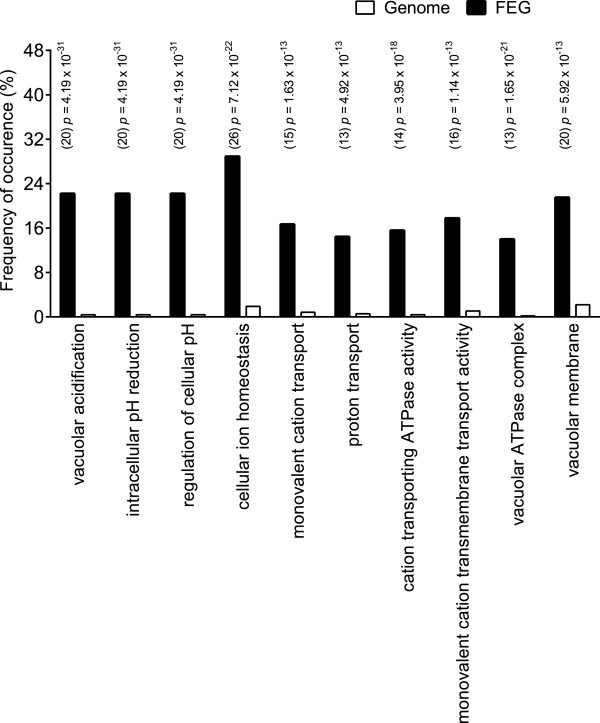
**Enrichment of FEG annotated to specific GO terms from SGD using GO ToolBox GO-Stats.** The frequency of gene occurrence (expressed as a percentage) for individual GO terms was compared between the 93 FEG dataset and the entire yeast genome (7168 genes). These GO terms were not mutually exclusive. The statistical probability was calculated to determine the enrichment of the specific GO terms, expressed as *p*-values. Numbers in brackets represent the number of genes in FEG annotated to a specific GO term. Data from the yeast genome is denoted with □ and the FEG dataset (93 genes) with ■.

A subset of the most enriched GO terms (*p* = 10^−31^ to 10^−13^) includes gene clusters related to vacuolar function and cellular pH and ion homeostasis (Figure 4). The vacuole has a multifunctional role; trafficking of membrane proteins to the plasma membrane, proteolytic degradation and recycling of proteins sent to the vacuole [32], sequestration of toxic metal ions, ion homeostasis (calcium and phosphate), osmo-regulation (Na^+^/K^+^ accumulation) and storage of amino acids [33]. Disruption of these functions can therefore have far reaching consequences for a cell grown at high osmolarity and low pH conditions.

### Incomplete ‘stuck’ fermentation as a result of deletion of specific genes

**Figure 5:**
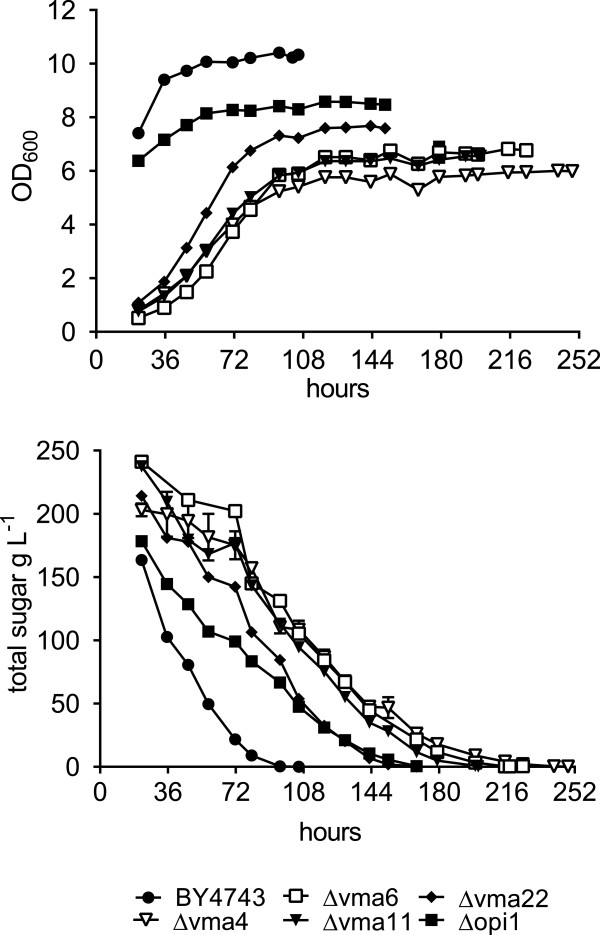
**Comparative fermentations with parent yeast BY4743 and four** Δ**vma mutants and** Δ**opi1 in CDGJM_200.** Fermentations (100 mL) were performed in triplicate in CDGJM_200 at 28°C whereby the deletants were compared directly with the parental strain BY4743. Fermentation progress was monitored by determining the total residual sugar (glucose and fructose) by enzymatic analysis. Growth was monitored as optical density (600 nm) 20 h post-inoculation and at intervals throughout fermentation.

Nine deletants within the 93 gene FEG dataset gave rise to incomplete (stuck) fermentations (Figure 2). These genes are central to ion homeostasis (*PTK2* (*YJR059W*)), *SSQ1* (*YLR369W*)), NAD recycling (*NPT1* (*YOR209C*)), signalling (*PLC1* (*YPL268W*)), trehalose synthesis (*TPS1* (*YBR126C*)*, TPS2* (*YDR074W*)), transcription (*SIN3* (*YOL004W*)*, ZAP1* (*YGR285C*)), and ubiquitin recycling (*DOA4* (*YDR069C*)). With the exception of Δtps1 and Δptk2, which showed normal growth, the other 7 ‘stuck’ mutants exhibited reduced biomass (as determined by maximum OD_600_). Our findings are only in part comparable to the SGD database where Δtps1, Δtps2, Δnpt1, Δplc1, and Δssq1 were shown to have reduced fermentative growth (Additional file 3 and Table 2). Underlying these differences is likely the experimental approach used. It is evident that final biomass (maximal OD_600_) does not necessarily determine whether yeast are able to catabolise sugar albeit in an extended time frame (protracted) or fail to catabolise all of the sugar (defined as a stuck fermentation). Four vma mutants (defective in vacuolar ATPase) that had a similar growth deficiency (plus an extended lag phase) were able to catabolise sugar albeit more slowly than the parent BY4743 (Figure 5).

**Table 2:** **SGD_FRYG fermentation phenotypes associated with nine ‘stuck fermentation’ mutants**

Acidic pH resistance	Hyperosmotic stress resistance	Oxidative stress resistance	Cold sensitivity	Heat sensitivity	Starvation resistance	Ethanol resistance
	*SSQ1*	*SSQ1*	*SSQ1*	*SSQ1*		*SSQ1*
*TPS2*	*TPS2*	*TPS2*		*TPS2*		*TPS2*
*DOA4*	*DOA4*	*DOA4*		*DOA4*		*DOA4*
	*PLC1*	*PLC1*		*PLC1*		*PLC1*
		*PTK2*			*PTK2*	*PTK2*
		*NPT1*		*NPT1*		
*TPS1*						*TPS1*
				*SIN3*		*SIN3*
*ZAP1*		*ZAP1*				

Phenotype responses which result in decreased resistance are denoted with a downward (↓) arrow, whilst those which result in increased sensitivity are denoted with an upward (↑) arrow.

All 9 genes associated with stuck fermentations were shown to have increased sensitivity towards multiple stresses relevant to fermentation (Table 2 and Additional file 3), with *DOA4, SSQ1* and *TPS2* being associated with five stresses. Interestingly, only *SIN3*, *SSQ1*, *TPS1* and *TPS2* genes were associated with ‘response to stress’ GO:0006950 (Additional file 4) although there was no over-representation within the FEG dataset for genes annotated to this GO term. Whilst Δplc1 results in decreased glucose utilisation, none of the 9 stuck mutants are listed as decreased fermentation metabolism, decreased nitrogen utilisation or decreased anaerobic growth (Additional file 3).

### Role of TPS1 and TPS2 as protectants against multiple stresses

*TPS1* (trehalose-6-phosphate synthase) and *TPS2* (trehalose-6-phosphate phosphatase) are required for trehalose biosynthesis and not surprisingly were both identified in this study as FEGs. Deletion of either *TPS1* or *TPS2* leads to complete cessation of sugar utilisation in the second half of fermentation i.e. a ‘stuck’ fermentation (Figure 2). Trehalose, a non-reducing disaccharide, ordinarily accumulates during stationary phase and is classified as a storage carbohydrate [34]. It is associated with increased survival and cell protection from stresses such as heat, ethanol and freezing [35]. Accordingly, trehalose protects against lipid peroxidation and protein carbonylation resulting from ethanol induced oxidative stress, which would otherwise impact on membrane dynamics and glycolysis, respectively [36]. Past studies have shown sensitivity of Δtps1 to ethanol [10] and oxidative stress [6], and Δtps2 to ethanol, heat, and NaCl [24], high acidity [25] and oxidative stress [6, 24]. Conversely, over-expression of these genes results in enhanced thermotolerance and improved ethanol resistance [35–37]. Given the recent link between mitochondrial mutation and ageing [38], and trehalose [36], and that fermentation is conducted by metabolically active but stationary phase cells, it is likely that the lack of trehalose in Δtps1 and Δtps2 [36] results in a vastly decreased ability of the cell to protect against the toxic effect of ethanol, leading to increased mitochondrial dysfunction and premature ageing and reduced viability during fermentation.

### Role of SIN3 in thermal stress

Adaptation to elevated temperatures, especially above 30°C and in the presence of ethanol [39], is fundamental to survival and the ability to complete fermentation. The primary mechanism is the heat shock response operating through chromatin modulation by the Rpd3L deacetylase complex, which is recruited to target promoters upon heat stress affecting transcription and general metabolic processes (reviewed in [40]). *SIN3* is associated with the heat shock response; deletion of which resulted in the complete arrest of fermentation (Figure 2). Sin3p is a component of the Rpd3L complex, along with Sap30p and Rxt3p. The latter two genes, when deleted, resulted in only protracted fermentation. The Rdp3L complex is proposed to facilitate the ability of yeast to tolerate multiple stresses including thermal and osmotolerance but not oxidative stress [28].

### SSQ1 and ZAP1 in ion homeostasis

Osmotic stress, whether salt or sugar induced, is well known to have a direct effect on cellular ion homeostasis [41]. In response to the external environment, various transporters transport cations into the organelles and across the plasma membrane, such that cell turgidity is maintained [42]. In this study 26 genes (28.9% of FEG genes) involved in ion homeostasis were identified as having a role in fermentation.

Two FEGs in particular, *SSQ1* and *ZAP1*, involved in iron and zinc homeostasis respectively, resulted in ‘stuck’ fermentation (Figure 2). *SSQ1* encodes a mitochondrial hsp70-type molecular chaperone that is required for the assembly of Fe-S clusters into Fe-S proteins, such as the mitochondrial proteins aconitase, Yfh1p and several DNA repair enzymes [43]. Iron homeostasis is proposed to be highly regulated, with the Fe-S clusters thought to function as regulatory sensors to oxidative stress and intracellular iron (reviewed in [43]). Yeast strains lacking the Fe-S cluster scaffold protein Isu1p or chaperone protein Ssq1p increase iron sequestration to the mitochondria, and show increased mitochondrial oxidative damage [43]. Transcription of *ISU1* was altered during fermentation in response to increasing external ethanol content [27], however, in this study Δisu1 had comparable fermentation duration to the parent (microferment 1; see Additional file 1). Given the speculation on Fe-S proteins acting as iron sensors, decreased formation of these proteins as observed in Δssq1 [44] may lead to a signalling of iron deficiency within the mitochondria. This may cause an increase in mitochondrial uptake and decrease in export of iron, and consequently mitochondrial iron overload and subsequent oxidative damage. Δssq1 has been previously shown to be sensitive to oxidation [44], which may explain the fermentation phenotype of Δssq1 in our study, in terms of oxidative stress associated with fermentation in CDGJM_200 and the low iron content (14 μg L^−1^) [17, 45], which is in the lower range (20–330 μg L^−1^) of typical Australian Chardonnay juices [46]. Given that mitochondrial dysfunction has recently been linked with vacuolar acidification in affecting chronological ageing [38] it is of interest that the Δssq1 deletant is not defective in vacuolar acidification ([47], data not shown).

It appears that zinc homeostasis is also critical to fermentation, through the transcriptional control of some 80 genes exerted by the zinc-sensing transcription factor Zap1p, (reviewed in [48]). These include genes involved in phospholipid biosynthesis, zinc uptake and vacuolar storage (detoxification), sulfur metabolism, cell wall function and response to oxidation (ROS) [48]. Zap1p has a protective role with regards to oxidative stress and damage. The down-regulation of sulfur metabolism by Zap1p in zinc deficient cells is associated with increased protection through the redirection of NADPH from sulfur metabolism to regenerating reduced peroxiredoxin and glutathione associated with antioxidant defence mechanisms (cited in [48]). The extended and incomplete fermentation profiles of Δzap1 (Figure 2) indicate that Zap1p’s role as a core regulatory protein particularly in oxidative protection and perhaps vacuolar storage is vital for fermentation maintenance.

### Influence of intracellular pH on fermentation

GO analysis of the FEG dataset (Additional file 4), not only highlighted the enrichment of 18 genes associated with the vacuolar H^+^ ATPase complex (targeted by Vph1p isomer of V_0_ subunit a) [49–51] but also *DBF2* (*YGR092W*) [52] and *NHX1* (*YDR456W*) [53], known to be involved with regulation of intracellular pH. The tight regulation of intracellular pH, whilst influenced by external pH and nutrient availability, is reflective of most physiological processes being pH dependent [48]. Critical to this, is the cytosol’s buffering capacity, originating from the acid–base action of metabolites such as ammonium, phosphate and carbon dioxide, as well as proteins [54]. As such, pH ‘sensing’ is associated with many processes including carbon flux through the glycolytic cycle, coupled reduction-oxidation reactions involved in NAD(P)^+^ cycling as well as phosphatidylinositol phosphates acting as signalling molecules to effect transcription [54].

The main pH regulator in yeast is a P2 type H^+^ATPase; Pma1p, responsible for the active translocation of protons across the plasma membrane into the external medium, thus maintaining cytosolic pH at neutral [48, 55]. The second, is the vacuolar (V) H^+^ATPase, a complex of two domains, V_1_ (8 subunits) and V_0_ (6 subunits), which is responsible for the active transport of protons from the cytosol into the vacuolar lumen [49]. *PMA1* (*YGL008C*) is an essential gene and so not present in the homozygous diploid deletion library used in this study. However, the heterozygous diploid (*PMA1*/Δ*pma1*) results in haploinsufficiency, i.e. reduced growth rate in grape juice conditions (202 mg L^−1^ YAN, 130 g L^−1^ water soluble carbohydrate (WSC)) and surprisingly also the reverse, haploproficiency (increased growth rate) in nitrogen and carbon limiting conditions (118.7 mg L^−1^ YAN, 2.5 g L^−1^ WSC) [56].

Pma1p activation is dependent on a complex interaction between glucose sensors such as Snf3p and Rgt2p, and the Gpr1p/Gpa2p receptor/G protein-coupled (GPCR) complex [57], protein kinase C (Pkc1p) [57], phospholipase C (Plc1p) [58], the proteosome, and interaction with tubulin (as reviewed in [54]). Several studies conducted on glucose starved cells, using specific inhibitors of Plc1 (3-nitrocourmarin) [59, 60] and Pma1p (carbonyl cyanide m-chlorophenylhydrazone; CCCP) [61] in conjunction with glucose additions (up to 100 mM or 18 g L^−1^) have demonstrated the activation of Pma1p to be mediated by Plc1p and the GPCR complex. Plc1p is proposed to transduce the glucose signal, through calcium signalling, by the induction of calcium uptake into the cell and vacuolar transport into the cytoplasm [59, 62–64] Gpr1p aids rapid adaption to extracellular glucose (and not fructose), via the cAMP-PKA pathway [65]. The latter activates cAMP signalling via a glucose phosphorylation mechanism [66].

In this study genes associated with hexose sensing were identified; *GPA2* (*YER020W*) and *GPR1* (*YDL035C)*), together with *ASC1* (*YMR116C)* and *HXK1* (*YFR053C*), whose deletion was shown to induce protracted fermentation (Additional files 1 and 4). Hxk1p, which is associated with heat shock response, is one of three protein kinases (Glk1p, Hxk1p, and/or Hxk2p) required for glucose phosphorylation, as a means of intracellular signalling via Gpr1p-Gpa2p. Whilst Asc1p, a guanine dissociation inhibitor of Gpa2p, links glucose metabolism to nitrogen metabolism, as it represses Gcn4p when amino acids are sufficient [66].

*PLC1 (YPL268W*) was also identified in the FEG dataset (Additional files 1 and 4). The inability of the Δplc deletant to complete sugar catabolism, as shown in Figure 2 alludes to the importance of Plc1p in cellular metabolism; although the physiological roles during fermentation are not well understood. Plc1p has been implicated in nutrient induced signal transduction via the Gpr1p-Gpa2p mediated GPCR system, coupling glucose and nitrogen signalling at the sensor level [64, 67], as well as controlling the MAP kinase cascade via activation of Pkc1p. This cascade is essential to adaptation to high osmolarity and cell wall integrity [41]; obvious important attributes for successful fermentation.

*HRK1* (*YOR267C*) and *PTK2* (*YJR059W*) were also identified and encode protein kinases responsible for the activation of Pma1p in response to glucose [68]. The membrane potential generated by Pma1p is modulated by the uptake of potassium (K^+^) by the Trk1p and Trk2p transporters; allowing for different voltage sensitive transporters to function in cation transport [48, 68]. The central role of pH (and cation) homeostasis in fermentation is further supported by the inclusion of *TRK1* (*YJL129C*) in the FEG dataset, which encodes the high affinity K^+^ transporter and is also found to be important for resistance to high-glucose [4] and high acidity [25].

Interestingly, Pma1p activity is reduced in yeast in high sugar fermentation conditions with recovery to normal activity being incomplete upon transfer to low sugar conditions [4]. Teixeira and co-workers [4] propose that vesicle trafficking to the plasma membrane is affected, given the mis-targeting of Pma1p in vma mutants lacking V-ATPase subunits to the vacuole [55]. pH and ion homeostatic mechanisms via V-ATPase are implicated in both osmotic shock from salt and sugar [4], with V-ATPase contributing to not only vesicle protein trafficking but intracellular (cytosolic) pH through the translocation of protons into the vacuolar lumen. *NHX1* (*YDR456W*) encoding the endosomal K^+^ (Na^+^)/H^+^ exchanger, Nhx1p, was also identified as a FEG in our study. Nhx1p is implicated in regulating vacuolar and cytosolic pH [55], and is thought to represent an early response to hyperosmotic conditions by the transport of ions into the vacuole [69].

The complex interaction between Pma1p, V-ATPase and other symporters is in part also mediated by the lipid rafts associated with these membrane bound proteins. In a recent study [70] lipid raft integrity of the plasma membrane was implicated in pH homeostasis and growth. Edelsofine, an anti-tumor lysophosphatidyl choline analog, was shown to alter the organisation of the plasma membrane, resulting in ubiquitination and internalisation of Pma1p to the vacuole, resulting in cytoplasmic acidification [70]. The link between membrane (lipid raft) integrity, intracellular pH, and fermentation phenotype is also inferred from the sensitivity of the following FEG mutants to Edelsofine, Δasc1, Δdfb2, Δsnf4, Δsin3, Δtrk1, Δvma2, Δvma4, Δvma5, Δvma6, Δvma7, Δvma10, Δvma11, Δvma16 and Δvph2 [70].

### Role of vacuolar H^+^ATPase in fermentation

As mentioned, 18 genes associated with vacuolar acidification, specifically the vacuolar ATPase complex were identified in this study (Additional file 4 and Table 3).

**Table 3:** **Effect upon fermentation of yeast lacking genes encoding vacuolar H** ^**+**^ **ATPase complex and associated proteins**

Gene	V-ATPase subunit	Function (eight subunit peripheral domain)	Essential for fermentation
	V _1_ domain		
*VMA1* (*TFP1*)	A	ATP hydrolysis	Yes
*VMA2*	B	Regulatory, ATP binding?, Actin binding?	Yes
*VMA5*	C	Stator	Yes
*VMA8*	D	Rotor	Yes
*VMA4*	E	Stator	Yes
*VMA7*	F	Rotor	Yes
*VMA10*	G	Stator	Yes
*VMA13*	H	Stator	Yes
	**V**_**o**_ **domain**	**(six subunit integral domain)**	
*VPH1*	a (vph1p)	Proton pore, stator, sorting (vacuole)	Yes
*STV1*	a (Stv1p)	Proton pore, stator, sorting (golgi/endosome)	No
*VMA3* (*CUP5*)	c	Proton pore, rotor (dicyclohexylcarbodiimide binding)	Yes
*VMA11* (*TFP3*)	c’	Proton pore, rotor	Yes
*VMA16* (*PPA1*)	c”	Proton pore, rotor	Yes
*VMA6*	d	Rotor	Yes
*VMA9*	e	?	nd
	**Assembly Factors**		
*VPH2* (*VMA12*)	Vph2p	Integral membrane protein; vacuolar H^+^ ATPase (V-ATPase) assembly	Yes
*VMA22*	Vma22p	Peripheral membrane protein; vacuolar H^+^ ATPase assembly	Yes
*VPS3*	Vps3p	CORVET tethering complex; cytoplasmic protein required for sorting & processing of soluble vacuolar proteins, acidification of vacuolar lumen, & assembly of V-ATPase	No
*PKR1*	Pkr1p	V-ATPase assembly factor, functions with other V-ATPase assembly factors in ER to assemble V-ATPase membrane sector (V_o_)	Yes
	**RAVE complex**		
*SKP1*	Skp1p	Evolutionarily conserved kinetochore protein; part of SCF ubiquitin ligase complex, CBF3 complex binding centromeric DNA, & RAVE complex regulating assembly of V-ATPase	nd
*RAV1*	Rav1p	Subunit of RAVE complex (Rav1p, Rav2p, Skp1p), promotes assembly of V-ATPase	Yes
*RAV2*	Rav2p	Subunit of RAVE complex, promotes assembly of V-ATPase	Yes

Fermentations (100 mL) were conducted in CDGJM_200 as described in Materials and Methods. Deletants which resulted in protracted fermentation were considered essential for fermentation in high sugar media, whilst those which were not affected, were considered non-essential.

*VMA9* (*YCL005W-A*) and *SKP*1 (*YDR328C*) are not in the homozygous diploid deletion library. *SKP*1 is an essential gene for growth.

nd: not determined.

The vacuolar ATPase complex is the major contributor in regulating organelle pH, specifically between the cytosol and vacuolar lumen [49]. This action is intrinsically connected to cellular ion homeostasis (through the action of anti-porters utilizing the generated membrane potential via a proton electrochemical gradient), as well as proteolytic activation (digestion of autophagasomes during autophagy or vacuolar proteolysis induced by starvation), endocytosis, and vacuole fusion [49, 71]. Furthermore, intracellular pH and Ca^2+^ ion concentrations as well as direct interaction of the V-ATPase complex with the actin cytoskeleton are implicated in bud formation and polarized growth during cell cycle [50, 72]. In relation to the vacuole’s lytic function, *VAM3* (*YOR106W*) encoding the syntaxin-like protein Vam3p (a vacuolar t-SNARE) is responsible for the correct trafficking and processing of proteinases A, B and carboxypeptidase Y and maturation of alkaline phosphatase [73]. The relevance of Vam3p to fermentation outcome and tolerance of stress (hyperosmolarity, heat, ethanol and oxidation) is highlighted by the protracted fermentation of the Δvam3 mutant (Additional file 1) and growth sensitivity towards these stress parameters (Additional file 2).

The importance of the assembly and function of the V-ATPase complex during fermentation, was highlighted by the number of genes identified (Additional files 4 and Table 3). These encode either the subunits of the V_1_ peripheral membrane and V_0_ integral membrane domains, or the assembly factors including the RAVE complex, which are required for the reversible assembly and disassembly into the free V_1_ and V_0_ domains, in response to glucose [50, 51]. The assembly of V-ATPase is thought to prevent cytosolic acidification when metabolism is active, as protons are sequestered in the vacuolar lumen by the V-ATPase. This is particularly relevant in high sugar fermentation, when the plasma membrane bound pump Pma1p, the principal regulator of cytosolic pH, is transiently inhibited [4]. As glucose becomes limited, the disassembly of some V-ATPase complexes is thought to conserve ATP, as ATP hydrolysis is absent in the isolated V_1_ domain. The role of vacuolar ATPase in fermentation is supported by previous research showing deletion of structural genes resulting in growth sensitivity to ethanol [8–10] and hyperosmolarity due to high concentrations of glucose (300 g L^−1^, [4]). How these differences relate to the pH-dependent growth of vma (vacuolar membrane ATPase) deletants [50] is unclear. Whilst vma deletants, through the loss of V-ATPase activity, exhibit growth sensitivity at high pH (>pH 7) or high extracellular calcium and zinc, and reportedly are unable to grow on non-fermentable carbon sources, they are able to grow under acidic conditions (pH 5.5) albeit slower than the wild type [50, 74]. In this study, the mutants were able to grow in CDGJM_200 (pH 3.5) after an extended lag period yet only reached 60-80% of the parent biomass (Figure 5). The ability of these cells to grow even in the presence of initially high sugar and acid and later, increasing ethanol, is strong evidence that vacuolar acidification by this enzyme complex is not the only mechanism involved. Yeast possess additional, and yet unidentified independent mechanisms to acidify the endosomal compartments including the vacuole. We have preliminary microscopy data using 6-carboxyfluorescein diacetate which supports this notion. Specifically, early in fermentation (24 h) vacuolar acidification, as anticipated, was reduced in a Δvma1 mutant compared to the parent BY4743. However, after 72 h and 125 h, by which time fermentation was progressing well, vacuolar acidification was restored in the mutant (data not shown). Recently, vacuolar acidification, particularly the proton dependent storage of neutral amino acids rather than protein degradation in the vacuole, has also been linked to chronological aging [38]. The authors proposed that age-induced mitochondrial dysfunction is a result of decreased vacuolar acidification in aging mother cells, and the regeneration of vacuolar acidification in budding daughter cells, is coincidental with rejuvenation of lifespan. This network between the vacuole and mitochondria, thought to be mediated by nutrient-sensing pathways such as PKA, Sch9 and TOR [38], is important given that fermentation occurs over an extended time-frame, primarily by stationary phase cells which are progressively aging.

Several proposed mechanisms to maintain vacuolar pH independent of V-ATPase include passive diffusion and dissociation of weak acids, endocytic internalisation of acid equivalents or Pma1p itself [74], and ammonium ions acting as protonophores after uptake [74]. These mechanisms would enable growth under acidic conditions through pH equilibration across the plasma membrane and vacuolar membrane, maintaining the intracellular pH gradient between the cytosol and vacuole, relative to the outside. This process is vital to cell adaption to fermentation associated stresses, and allowing for complete sugar catabolism albeit at a slower rate. Whilst some of these alternative mechanisms may play a role normally in pH regulation, V-ATPase is the principal proton pump proposed to protect yeast from heat stress [75], alcohol stress [8, 9], osmotic stress [4], and acid stress [25] associated with fermentation, as well as against stresses of air-drying [29]. The latter is important in this context since commercial wine yeast is generally supplied as active dried yeast.

Whilst there are a myriad of processes and signalling cascades related to stress response, it is only recently that the inter-relationships are being elucidated. For example V-ATPase and the high-osmolarity glycerol (HOG) response pathway are proposed to function in parallel, as adaptive mechanisms to prevent salt toxicity under salt induced stress [76]. Both pathways are crucial for successful sugar fermentation, with not only vma mutants being identified but also the genes for Hog1p and its activator, Pbs2p. It is possible that communication between such processes as vacuolar acidification (V-ATPase), protein trafficking and turnover, and the TOR and HOG signalling pathways is through inositol-containing lipids in stress response signaling [77], in response to stress adaptation.

### Microautophagy and the EGO/GSE complex and fermentation

*MEH1* and *SLM4* encode 2 of the 3 subunits of the EGO complex and deletion of either severely affected fermentation in this study (65% slower than the parent). With the third component, Gtr2p, the EGO complex is a vacuolar membrane-associated protein complex required for activation of microautophagy [78] through its function as an activator of the nitrogen regulatory TORC1 (Tor1p or Tor2p-Kog1p-Lst8p-Tco89p) complex [79]. In this study *TCO89* was also identified as a FEG, with Δtco89 increasing fermentation duration by 43%. Meh1p and Slm4p are also members of the GSE (GAP1 Sorting in the Endosomes) complex, a GTPase complex required for intracellular sorting of Gap1p out of the endosome, for eventual delivery to the plasma membrane [80]. The GSE complex is also composed of two small GTPases (Gtr1p and Gtr2p) and Ltv1p, which do not have a critical role in fermentation (Additional file 1). These findings suggest that *MEH1* and *SLM4* have a major role in fermentation, through vacuolar acidification (Meh1p), probably through amino acid uptake into the vacuole, microautophagy and protein sorting of Gap1p.

Microautophagy and the EGO/GSE complex have been previously implicated in mechanisms allowing yeast cells to grow under low temperature and high pressure [7]. Abe and Minegishi [7] proposed that the EGO/GSE complex may contribute to cell surface delivery of amino acid permeases under such conditions, given the marked defect in histidine, leucine and lysine uptake in the EGO/GSE complex mutants (Δgtr1, Δgtr2, Δmeh1 (Δego1) and Δslm4 (Δego3)), upon high pressure and low temperature incubation.

As mentioned, the EGO complex is involved in nitrogen sensing via its activation of the TORC1 complex, such that EGO monitors intracellular levels of leucine and glutamine (reviewed in [79]). The inability to activate TORC1 during fermentation may result in fermentation arrest via several processes in which TORC1 is involved: the regulation of amino acid uptake, early glycolysis or induction of autophagy (reviewed in [79]).

It is known that autophagy is triggered by nutritional stress, specifically carbon and nitrogen depletion, and is induced as a prelude to autolysis in yeast conducting the secondary fermentation of sparkling wine production [81]. Autophagy is critical for fermentation outcome in primary fermentation as shown in our study, with 13 related genes identified as FEGs. In a recent study, Piggott et al. [82] looked at competitive fitness (growth) during a 12-day fermentation of a deletion library pool (S288c) in synthetic grape juice. Autophagy and ubiquitination were enriched in the dataset with respect to reduced fitness. However, our datasets for these GO terms are very different, with only 3 genes in common for autophagy; (*ATG7* (*YHR171W*), *SNX4/ATG24* (*YJL036W*), *CIS/ATG31* (*YDR022C*)). Other contradictions were the observations for Δdoa4 and Δpex1. Deletion of *DOA4* (homozygous diploid) did not result in reduced competitive fitness [82], contrary to our findings, wherebyΔdoa4 resulted in stuck fermentation. Another anomaly was the finding that Δpex1 (protracted fermentation in our study) resulted in increased fitness [82], implying that fermentation would be either similar to the parent or faster. The recent revelation that autophagy occurs not only during starvation but very early in fermentation [82], in response to as yet unknown stresses, alludes to the importance of the various autophagic responses and would explain the observed protracted fermentation phenotype in such mutants in our study.

It is evident that a number of mechanisms involving a myriad of genes are involved in the adaptive response to the major stressors encountered during high sugar fermentation as such only some of these have been discussed to in this study.

## Conclusion

This study reports on the identification of 93 genes whose deletion leads to fermentations that are protracted (84 genes) or arrested ("stuck"; 9 genes). Together, the 93 genes are referred to as *F*ermentation *E*ssential *G*enes, and are representative of the ‘fermentome’; a dataset of genes (from laboratory yeast) which modulate fermentation. The importance of the FEG is further supported through their presence in two fermentation relevant databases, LIT_FRYG and SGD_FRYG (Additional file 3), collated as part of this study. These datasets are by no means complete, but will be expanded upon as new datasets become available.

In this study we have built upon the understanding of a group of key genes within fermentation. We have identified several cellular processes essential for response and adaptation to a physiologically stressful environment in yeast, namely maintenance of vacuolar acidification, microautophagy and sugar related signalling. Whilst individual biological processes are alluded to with respect to fermentation, the question remains; how do these different sensors, signals and cellular responses interact with each other? A systems biology approach allows us to look at what appear to be unrelated processes, together as a ‘network’ or ‘matrix’, in a holistic approach, as well as the traditional reductionist approach [2, 19]. By taking such a perspective on wine fermentation, we have gained a better understanding of a fundamental biochemical pathway. This understanding allows design of new industrial yeast strains better suited to specific industrial processes. For example demonstration for the importance of ion homeostasis and intracellular pH maintenance raises the possibility that Directed Evolution could be used to generate new strains using a low pH environment as the selective pressure.

## Methods

### Yeast strains and media

This study used the collection of yeast diploid deletion strains developed by the Yeast Genome Deletion Project [12]. The homozygous (Δ*orf::KanMX4*) gene deletions were harboured in BY4743 (*MATa/a*, *his3*Δ*1/his3*Δ*1, leu2*Δ*0/leu2*Δ*0, lys2*Δ*0/LYS2, MET15/met15*Δ*0, ura3*Δ*0/ura3*Δ*0* (BY4741/BY4742)). Strains were maintained on YPD (1% yeast extract, 2% bactopeptone, 2% D-glucose) containing 200 mg L^−1^ geneticin (G418 sulfate; Amresco).

Chemically defined grape juice medium containing 200 g L^−1^ sugar as equimolar amounts of glucose and fructose (high sugar; CDGJM_200) and 450 mg L^−1^ FAN as a mixture of amino acids and ammonium chloride [17, 45] was used. The CDGJM_200 was supplemented with 150 mg L^−1^ uracil, and included 3 g L^−1^ polyphenol extract (Cat: Tppr, OenoProd, Sarl), which was dissolved in 100% ethanol (3 g powder in 5 mL, 24 h, in dark) prior to being added to the sterile medium. The CDGJM _200 was stored in the dark for a maximum of 24 h (4°C) before use. CDGJM_20 was identical to CDGJM_200 except that the sugar concentration was decreased to 20 g L^−1^ (low sugar; CDGJM_20).

### Genome-wide screening for yeast deletants with protracted fermentation in high sugar media (CDGJM_200)

Liquid handling of the samples was performed using a CAS3800 robot (Corbett Robotics, Sydney). The library was replicated (6 μL) into 0.6 mL YPD in 96 deep-well plates (Cat: P-DW-20-C, Pacific Lab Products) and incubated 24–48 h at 28°C in a humidified box without shaking. Each plate had 11 rows (A1-11 to H1-11) containing single samples of the deletion strains and four replicates each of the parental strain, BY4743 (A12-D12) and un-inoculated controls, CDGJM_200 (E12-H12). Cells were resuspended by agitation on a mini-vortexing-shaker and 6 μL was inoculated into 0.6 mL CDGJM_200 in 96 deep-well plates and covered with breathable sealing film (Cat: BF-400, Adelab Scientific). The initial fermentation screen was performed in duplicate in humidified boxes. After 180 h at 28°C, the cells were pelleted by centrifugation (1620 x g, 5 min) and 0.2 mL supernatant samples were dispensed into 96 well flat bottomed plates (Cat: P-96-300 F-PS, Adelab Scientific) and frozen at −20°C for subsequent sugar analysis. Residual glucose and fructose were quantified enzymatically using a commercially available kit (Cat: 139106, Boehringer Mannheim) adapted for 96-well plates. The total volume of the reaction was reduced from 1.62 mL to 0.125 mL. Three times the concentration of the reaction buffer was used to provide NADP and ATP to excess, and to ensure accurate pipetting, 10 μL of a 1:10 dilution of the enzymes in MQ water was used (as opposed to 1 μL). The samples were diluted 1:10 (for < 20 g L^−1^) and 1:100 (for > 20 g L^−1^) and determined against a set of identically diluted standards which were within the linear range of the reaction (0–0.08 g L^−1^). Absorbance at 340 nm was measured using a μQuant microplate spectrophotometer (Bio-Tek Instruments).

A second experiment (*cf* initial screen) was conducted, using a sacrificial plate method [83] on the 336 deletion strains identified as having protracted fermentation. At specific time points during fermentation (41, 89, 113, 137 and 190 h), a replicate plate was sacrificed to measure growth (OD_600_) and residual sugar in the CDGJM_200 media. Absorbance was measured using a microplate spectrophotometer.

Low sugar fermentations were also conducted on the 336 deletants (cf second screen) using CDGJM_20, in which the sugar content was reduced to 20 g L^−1^ and the duration of the fermentation was 56 h. Growth and fermentation progress was monitored as previously described at two time points: 32 and 56 h.

### Evaluation of identified yeast deletants in laboratory scale (100 mL) fermentations in CDGJM_200

Triplicate 100 mL fermentations were performed in CDGJM_200 using 250 mL Erlenmeyer flasks fitted with airlocks [84]. N_2_ sparging was omitted as previous studies demonstrated that anaerobic conditions were achieved within 4–6 hours of fermentation commencement (data not shown). Fermentation progress was monitored by refractive index (°Brix). Fermentations were considered dry and terminated when the residual sugar was less than 2.5 g L^−1^ as determined by Clinitest™ (Cat: 2107, Bayer). Residual glucose and fructose were determined by enzymatic analysis as described above.

### Confirmation of the identity of yeast deletants

Gene deletions in the 93 FEG (Fermentation Essential Genes) yeast clones were confirmed by PCR amplification of deletion cassettes from isolated genomic DNA using the protocol described in the *Saccharomyces* Genome Deletion Project (http://www-sequence.stanford.edu/group/yeast_deletion_project/deletions3.html). The PCR products of select clones were verified by DNA sequencing (AGRF, Adelaide).

### Classification and numeric enrichment of identified genes annotated to Gene Ontology (GO) terms using computational software tools

The datasets were analysed using GOToolBox; specifically GO-Stats (http://genome.crg.es/GOToolBox/) [30] and GO Finder Version 0.83 software (http://www.yeastgenome.org/cgi-bin/GO/goTermFinder.pl) which allowed the hierarchical clustering and over-representation (enrichment) or under-representation (depletion) of genes based on shared Gene Ontology (GO) terms. Both computational tools use a hypergeometric distribution with Multiple Hypothesis (Bonferroni) Correction (and additional *F*alse *D*iscovery *R*ate; GO Finder) to calculate *p*-values. The clustering of genes based on their shared gene annotations was also determined using GO-Proxy (http://genome.crg.es/GOToolBox/) and/or SGD Gene Ontology Slim Mapper (http://www.yeastgenome.org/cgi-bin/GO/goSlimMapper.pl).

The interpretation of the GO terms identified was simplified using the web-based visualisation tool GO-*Module* (http://lussierlab.org/GO-module) [31]. GO biomodules are ‘nodes’ which represent the key GO terms (K nodes) and their hierarchical descendents which are considered true-positive GO terms (T nodes). False positive prioritized GO terms in the input data are identified as F nodes.

Two databases denoted **F**ermentation **R**elevant **Y**east **G**enes (FRYG), were compiled from data available on the *Saccharomyces* Genome Database (http://www.yeastgenome.org/) and from a literature survey. The databases referred to as SGD_FRYG and LIT_FRYG were used in comparison studies. Statistical analysis of the results was performed using a hyper geometric algorithm as described by Martin et al. [30] to determine whether there was an enrichment (over representation) of genes identified in this study when compared to the FRYG datasets.

### Availability of supporting data

The data set supporting the results of this article is available in the LabArchives repository, under Fermentation Microbiology doi:10.6070/H4057CW1 and hyperlink to dataset(s) in https://mynotebook.labarchives.com/share/Fermentation%2520Microbiology/MjQuN3w0MDY2Ni8xOS9UcmVlTm9kZS8xMzY5ODkyMTYxfDYyLjc=.

The data set supporting the results of this article is included within the article (and its additional file(s)).

## Electronic supplementary material

Additional file 1: **Experimental data from micro-scale and laboratory-scale fermentations in chemically defined grape juice medium.** Micro-fermentation screens 1 and 2 were conducted in high sugar media (CDGJM_200) whilst micro-fermentation 3 was conducted in a low sugar medium (CDGJM_20). Values were the average of duplicate fermentation samples per strain. Laboratory scale (100 mL) fermentations were performed in CDGJM_200, and the values represent the average of triplicate fermentations. Data for optical density measurements are not included. (XLSX 1 MB)

Additional file 2: **Summary of fermentation data for yeast deletants compared to parent BY4743.** CDGJM_200 100 mL: Yeast deletants were compared with parent BY4743 in triplicated 100 ml fermentations using CDGJM_200. The degree of protraction was determined as the fermentation duration ratio of the deletant compared to BY4743. 72 genes were identified as effecting fermentation. Durations of 120% and above were considered significant in exhibiting protracted fermentation. An additional 29 genes were examined, 21 of which were shown to result in protracted fermentation in CDGJM_200. CDGJM_20 micro: Yeast deletants were compared with parent BY4743 in micro-scale fermentations using CDGJM_20. 101 clones were examined of which 72 were protracted in fermentation using CDGJM_200. Residual sugar and OD_600_ was determined at 32 h and 56 h. (XLSX 48 KB)

Additional file 3: **Comparison of the FEG dataset with the Fermentation Relevant Yeast Gene Databases LIT_FRYG and SGD_FRYG.** Additional file 2 is a Microsoft Office Excel workbook relating to analysis of the genes identified in this study compared to previous research findings. Ninety three genes representing the FEG dataset were identified. Deletion of these genes resulted in protracted fermentation (20% and greater when compared to parent, BY4743). See Additional file 1 for fermentation duration values. Two complementary databases of yeast genes related to fermentation, referred to as FRYG (acronym for Fermentation Relevant Yeast Genes) were generated. The first (LIT_FRYG) was compiled from relevant literature, examining single stress conditions relevant to fermentation. The second (SGD_FRYG), a compilation of phenotype terms as accessed on the SGD, whereby only null mutants (deletants) with a specific phenotype response, i.e. decreased resistance were selected. The 93 FEG were compared to the two FRYG datasets. Genes unique to this fermentation study (absent in the LIT_FRYG and SGD_FRYG) and two specific subsets of genes within the 93 FEG dataset (“Stuck mutants” and “vacuolar (H^+^) ATPase”) are shown. (XLSX 236 KB)

Additional file 4: **Classification and numerical enrichment of the FEG annotated to biological process, function, and cellular component.** Genes were classified using the computational tools Slim Mapper (SGD database; (http://www.yeastgenome.org/) and GOToolBox into categories based on GO terms for biological process, function and cellular component. Descriptions of the 93 FEG are taken from SGD database. Enrichment of genes annotated to particular GO terms includes corresponding *p* values. GO terms were organised into GO biomodules using the web based visualisation tool GO-Module whereby K nodes refer to the key GO terms and T nodes refer to hierarchical descendents of K, regardless of statistical value (*p* value). F nodes represent the false positive prioritised GO terms in the input data. F nodes determined for particular GO terms were not precluded because of the nature of the GO term. Genes annotated to GO terms not defined through GO module and not significantly enriched were classified into groups using SGD Slim Mapper. (DOCX 48 KB)
