# Supplementary material for: Genome-wide identification of the Fermentome; genes required for successful and timely completion of wine-like fermentation by Saccharomyces cerevisiae
Source: BMC Genomics. 2014 Jul 3;15(1):552. doi: 10.1186/1471-2164-15-552 (PMC4099481; doi:10.1186/1471-2164-15-552)
Supplement: Supplementary file 4 — Additional file 4: Classification and numerical enrichment of the FEG annotated to biological process, function, and cellular component. Genes were classified using the computational tools Slim Mapper (SGD database; (http://www.yeastgenome.org/) and GOToolBox into categories based on GO terms for biological process, function and cellular component. Descriptions of the 93 FEG are taken from SGD database. Enrichment of genes annotated to particular GO terms includes corresponding p values. GO terms were organised into GO biomodules using the web based visualisation tool GO-Module whereby K nodes refer to the key GO terms and T nodes refer to hierarchical descendents of K, regardless of statistical value (p value). F nodes represent the false positive prioritised GO terms in the input data. F nodes determined for particular GO terms were not precluded because of the nature of the GO term. Genes annotated to GO terms not defined through GO module and not significantly enriched were classified into groups using SGD Slim Mapper. (DOCX 48 KB) [file 12864_2013_6243_MOESM4_ESM.docx]

| **Ferment Duration Ratio** | **GO:ID** | **GO Term** | **Frequency in genome** | **Frequency in FEG** | | | **Enrichment (*p*-value)** | **GO-module node** | | **Genes and Description** |
| --- | --- | --- | --- | --- | --- | --- | --- | --- | --- | --- |
| *0007035* | | *vacuolar acidification* | *0.0039* | *0.2222* | | | *4.19E-31* | *K* | | *TFP3,VMA5,VMA22,DBF2,MEH1,VMA2,NHX1,VMA13,*  *VMA10,VMA4,VMA7,VPH1,PPA1,CUP5,TFP1,VMA6,*  *RAV1,RAV2,VPH2,VMA8* |
| *0051452* | | *intracellular pH reduction* | *0.0039* | *0.2222* | | | *4.19E-31* | *T* | |  |
| *0051453* | | *regulation of intracellular pH* | *0.0041* | *0.2222* | | | *1.79E-30* | *T* | |  |
| *0030641* | | *regulation of cellular pH* | *0.0041* | *0.2222* | | | *1.79E-30* | *T* | |  |
| *0006873* | | *cellular ion homeostasis* | *0.0189* | *0.2889* | | | *7.12E-22* | *K* | | *TFP3,VMA5,VMA22,ZAP1,PTK2,DBF2,HRK1,MEH1,*  *VMA2,NHX1,SSQ1,VMA13,VMA10,VMA4,VMA7,VPH1,*  *OCT1,TRK1,PPA1,CUP5,TFP1,VMA6,RAV1,RAV2,VPH2,VMA8* |
| *0030003* | | *cellular cation homeostasis* | *0.0165* | *0.2667* | | | *1.46E-20* | *T* | | *TFP3,VMA5,VMA22,ZAP1,DBF2,MEH1,VMA2,NHX1,*  *SSQ1,VMA13,VMA10,VMA4,VMA7,VPH1,OCT1,TRK1,*  *PPA1,CUP5,TFP1,VMA6,RAV1RAV2,VPH2,VMA8* |
| *0015672* | | *monovalent inorganic cation transport* | *0.0083* | *0.1667* | | | *1.63E-13* | *K* | | *TFP3,VMA5,VMA2,NHX1,VMA13,VMA10,VMA4,VMA7,*  *VPH1,TRK1,PPA1,CUP5,TFP1,VMA6,VMA8* |
| *0015992* | | *proton transport* | *0.0057* | *0.1444* | | | *4.92E-13* | *T* | | *TFP3,VMA5,VMA2,VMA13,VMA10,VMA4,VMA7,VPH1,*  *PPA1,CUP5,TFP1,VMA6,VMA8* |
| *0016471* | | *vacuolar proton-transporting V-type ATPase complex* | *0.0024* | *0.1444* | | | *7.42E-21* | *K* | |  |
| *0005774* | | *vacuolar membrane* | *0.0186* | *0.2222* | | | *1.46E-14* | *K* | | *TFP3,VMA5,VAM3,SLM4,MEH1,VMA2,VPS41,VMA13,*  *VMA10,VMA4,VMA7,VPH1,PPA1,TCO89,CUP5,TFP1,*  *VMA6,RAV1,VAC8,VMA8* |
| *0005773* | | *vacuole* | *0.0371* | *0.2333* | | | *1.14E-09* | *K* | | *TFP3,VMA5,VAM3,SLM4,MEH1,VMA2,VPS41,VMA13,*  *VMA10,VMA4,VMA7,VPH1,PPA1,TCO89,CUP5,TFP1,*  *VMA6,RAV1,CCZ1,VAC8,VMA8* |
| *0006119* | | *oxidative phosphorylation* | *0.0091* | *0.1333* | | | *1.16E-08* | *K* | | *TFP3,VMA5,VMA2,VMA13,VMA4,VMA7,VPH1,PPA1,*  *CUP5,TFP1,VMA6,VMA8* |
| *0070072* | | *vacuolar H^+^ATPase complex assembly* | *0.0006* | *0.0333* | | | *8.12E-03* | *K* | | *VMA22,PKR1,VPH2* |
| *0043291* | | *RAVE complex* | *0.0005* | *0.0222* | | | *0.112747* | *F* | | *RAV1,RAV2* |
| 1.7 | *CCZ1* | *YBR131W* | Protein involved in vacuolar assembly; autophagy and cytoplasm-to-vacuole pathway | | | | | | | |
| 2.4 | *CUP5* | *YEL027W* | *VMA3*; Proteolipid subunit of vacuolar H^+^ ATPase Vo sector (subunit c; dicyclohexylcarbodiimide binding subunit); vacuolar acidification and copper and iron metal ion homeostasis | | | | | | | |
| 1.4 | *DBF2* | *YGR092W* | Ser/Thr kinase involved in transcription and stress response; localization is cell cycle regulated | | | | | | | |
| 2.9 | *HRK1* | *YOR267C* | Protein kinase implicated in activation of plasma membrane H^+^ ATPase Pma1p in response to glucose metabolism; role in ion homeostasis | | | | | | | |
| 1.7 | *MEH1* | *YKR007W* | Component of EGO complex, involved in regulation of microautophagy, and of GSE complex, required for sorting of amino acid permease Gap1p; loss results in a defect in vacuolar acidification | | | | | | | |
| 1.7 | *NHX1* | *YDR456W* | Na^+^/H^+^ and K^+^/H^+^ exchanger; intracellular sequestration of Na^+^ and K^+^; located in vacuole and late endosome; required for osmotolerance to acute hypertonic shock and vacuolar fusion | | | | | | | |
| 1.4 | *OCT1* | *YKL134C* | Mitochondrial intermediate peptidase; may contribute to mitochondrial iron homeostasis | | | | | | | |
| 1.2 | *PKR1* | *YMR123W* | V-ATPase assembly factor, functions with other assembly factors in ER to assemble V-ATPase membrane sector (V_0_) | | | | | | | |
| 3.4 | *PTK2* | *YJR059W* | Putative serine/threonine protein kinase involved in regulation of ion transport across plasma membrane; enhances spermine uptake | | | | | | | |
| 2.5 | *RAV1* | *YJR033C* | Subunit of RAVE complex (Rav1p, Rav2p, Skp1p), promotes assembly of H^+^ ATPase holoenzyme; required for transport between early and late endosome/PVC and localization of TGN membrane proteins | | | | | | | |
| 1.8 | *RAV2* | *YDR202C* | Subunit of RAVE complex, associates withV_1_ domain of vacuolar H^+^ ATPase, promotes assembly and reassembly of holoenzyme | | | | | | | |
| 5.2 | *SSQ1* | *YLR369W* | Mitochondrial hsp70-type molecular chaperone, required for assembly of iron/sulfur clusters into proteins after cluster synthesis | | | | | | | |
| 1.4 | *TCO89* | *YPL180W* | Subunit of TORC1 complex, regulates growth response to nutrient availability; cooperates with Ssd1p in cellular integrity maintenance | | | | | | | |
| 2.9 | *TFP1* | *YDL185W* | *VMA1*; subunit A of V_1_ domain of vacuolar H^+^ ATPase; protein precursor undergoes self-catalyzed splicing to yield extein Tfp1p and intein Vde (PI-SceI), a site-specific endonuclease | | | | | | | |
| 2.3 | *TFP3* | *YPL234C* | VMA11; vacuolar H^+^ ATPase V_0_ domain subunit c', involved in proton transport activity; hydrophobic integral membrane protein (proteolipid) containing four transmembrane segments; N and C termini are in the vacuolar lumen | | | | | | | |
| 1.4 | *TRK1* | *YJL129C* | Component of Trk1p-Trk2p potassium transport system; 180 kDa high affinity potassium transporter | | | | | | | |
| 2.0 | *VMA2* | *YBR127C* | Subunit B of V_1_ domain of vacuolar H^+^ ATPase , an electrogenic proton pump; contains nucleotide binding sites, found in cytoplasm | | | | | | | |
| 2.4 | *VMA4* | *YOR332W* | Subunit E of the V_1_ domain of the vacuolar H^+^ ATPase (V-ATPase); V-ATPase is an electrogenic proton pump found throughout the endomembrane system; V_1_ domain has eight subunits; required for the V_1_ domain to assemble onto the vacuolar membrane; protein abundance increases in response to DNA replication stress | | | | | | | |
| 2.7 | *VMA5* | *YKL080W* | Subunit C of V_1_ domain of vacuolar H^+^ ATPase; V_1_ domain assembly onto vacuolar membrane | | | | | | | |
| 2.1 | *VMA6* | *YLR447C* | V_0_ domain subunit d of vacuolar H^+^ ATPase, an electrogenic proton pump found in the endomembrane system; stabilizes V_0_ subunits; required for V_1_ domain assembly on the vacuolar membrane | | | | | | | |
| 1.9 | *VMA7* | *YGR020C* | Subunit F of V_1_ domain of vacuolar H^+^ ATPase; V_1_ domain assembly onto vacuolar membrane | | | | | | | |
| 1.7 | *VMA8* | *YEL051W* | Subunit D of V_1_ domain of vacuolar H^+^ ATPase; coupling of proton transport and ATP hydrolysis | | | | | | | |
| 2.7 | *VMA10* | *YHR039C-A* | Subunit G of V_1_ domain of vacuolar H^+^ ATPase; involved in vacuolar acidification | | | | | | | |
| 2.6 | *VMA13* | *YPR036W* | Subunit H of V_1_ domain of vacuolar H^+^ ATPase; serves as activator or structural stabilizer of V-ATPase | | | | | | | |
| 1.7 | *VMA16* | *YHR026W* | *PPA1*; Subunit c'' of vacuolar H^+^ATPase; vacuole acidification; one of three proteolipid subunits of V_0_ domain | | | | | | | |
| 1.6 | *VMA22* | *YHR060W* | Peripheral membrane protein that is required for vacuolar H^+^ ATPase (V-ATPase) function, although not an actual component of the V-ATPase complex; functions in the assembly of the V-ATPase; localized to the yeast endoplasmic reticulum (ER) | | | | | | | |
| 1.8 | *VPH1* | *YOR270C* | Subunit a of vacuolar H^+^ATPase V_0_ domain; two isoforms: Vph1p in vacuolar V-ATPase and Stv1p in V-ATPase of Golgi and endosomes | | | | | | | |
| 1.5 | *VPH2* | *YKL119C* | Integral membrane protein required for vacuolar H^+^ ATPase assembly, not component of the V-ATPase complex; endoplasmic reticulum (ER) | | | | | | | |
| 3.9 | *ZAP1* | *YJL056C* | Zinc-regulated transcription factor; binds to zinc-responsive promoters, induces gene transcription of presence of zinc, and repression in low zinc | | | | | | | |
| *0006796* | | *phosphate-containing compound metabolic process* | *0.0482* | *0.2* | | | *1.17-02* | *T* | *TFP3,VMA5,PTK2,DBF2,HRK1,VHS1,VMA2,VMA13,*  *VMA4,VMA7,HOG1,VPH1,PPA1,CUP5,TFP1,VMA6,*  *PBS2,VMA8* | |
| 1.2 | *VHS1* | *YDR247W* | Cytoplasmic serine/threonine protein kinase; suggested role in G1/S phase progression | | | | | | | |
| 2.1 | *HOG1* | *YLR113W* | Mitogen-activated protein kinase involved in osmoregulation via three osmosensors; recruitment and activation of RNA Pol II at Hot1p-dependent promoters; localization regulated by Ptp2p and Ptp3p | | | | | | | |
| 2.3 | *PBS2* | *YJL128C* | MAP kinase kinase, pivotal role in osmosensing signal-transduction pathway, activated under severe osmotic stress; regulates Ty1 transposition | | | | | | | |
| *0010255* | | *glucose mediated signaling pathway* | *0.0008* | *0.0333* | | | *2.00E-02* | *K* | *GPA2,GPR1,ASC1* | |
| *0009746* | | *response to hexose stimulus* | *0.0016* | *0.0333* | | | *2.24E-01* | *K* |  |  |
| *0007166* | | *cell surface receptor signaling pathway* | *0.0091* | *0.0556* | | | *8.54E-01* | *K* | *GPA2,GPR1,HOG1,ASC1,PBS2* | |
| 1.2 | *ASC1* | *YMR116C* | G-protein beta subunit and guanine nucleotide dissociation inhibitor for Gpa2p; small (40S) ribosomal subunit; represses Gcn4p in absence of amino acid starvation | | | | | | | |
| 1.3 | *GPA2* | *YER020W* | Nucleotide binding alpha subunit of heterotrimeric G protein, interacts with receptor Gpr1p, signaling role in response to nutrients | | | | | | | |
| 1.2 | *GPR1* | *YDL035C* | Plasma membrane G protein coupled receptor (GPCR) interacts with Gpa2p and Plc1p; sensor integrates nutritional signals with cell fate via PKA and cAMP synthesis | | | | | | | |
| *0016237* | | *microautophagy* | *0.0068* | *0.0889* | | | *8.13E-05* | *K* | | *VAM3,SLM4,MEH1,ATG7,VPS41,SNX4,CIS1,VAC8* |
| *0034727* | | *piecemeal microautophagy of nucleus* | *0.0052* | *0.0667* | | | *3.95E-03* | *T* | | *VAM3,ATG7,VPS41,SNX4,CIS1,VAC8* |
| *0000422* | | *mitochondrion degradation* | *0.0055* | *0.0667* | | | *5.63E-03* | *K* | |  |
| *0006914* | | *autophagy* | *0.0389* | *0.1444* | | | *2.27E-02* | *T* | | *VAM3,SLM4,DCC1,MEH1,GPR1,ATG7,VPS41,SNX4,*  *RXT3,CIS1,CCZ1,VAC8,XRS2* |
| *0005770* | | *late endosome* | *0.0035* | *0.0444* | | | *4.14E-02* | *K* | | *SLM4,MEH1,NHX1,CCZ1* |
| *0005768* | | *endosome* | *0.0195* | *0.0889* | | | *5.45E-02* | *T* | | *SLM4,MEH1,NHX1,VPS41,SNX4,DOA4,RAV2,CCZ1* |
| *0034448* | | *EGO complex* | *0.0005* | *0.0222* | | | *0.112747* | *F* | | *SLM4,MEH1* |
| *0034449* | | *GSE complex* | *0.0008* | *0.0222* | | | *0.365479* | *F* | |  |
| 1.5 | *ATG7* | *YHR171W* | Autophagy-related protein and dual specificity member of E1 family of ubiquitin-activating enzymes; mediates conjugation of Atg12p with Atg5p and Atg8p with phosphatidylethanolamine, required steps in autophagosome formation | | | | | | | |
| 1.3 | *CIS1* | *YDR022C* | Autophagy-specific protein required for autophagosome formation; may form complex with Atg17p and Atg29p localising other proteins to pre-autophagosomal structure | | | | | | | |
| 1.4 | *DCC1* | *YCL016C* | Subunit of complex with Ctf8p and Ctf18p, required for sister chromatid cohesion and telomere length maintenance | | | | | | | |
| 2.5 | *DOA4* | *YDR069C* | Ubiquitin isopeptidase, involved in recycling ubiquitin from proteasome-bound ubiquitinated intermediates | | | | | | | |
| 1.8 | *RXT3* | *YDL076C* | Subunit of RPD3L complex; involved in histone deacetylation | | | | | | | |
| 1.7 | *SLM4* | *YBR077C* | Component of EGO complex, involved in regulation of microautophagy, and of GSE complex, required for amino acid permease Gap1p sorting | | | | | | | |
| 1.2 | *SNX4* | *YJL036W* | Sorting nexin, involved in retrieval of late-Golgi SNAREs from post-Golgi endosomes to trans-Golgi network and in cytoplasm to vacuole transport; forms complexes with Snx41p and Atg20p | | | | | | | |
| 1.5 | *VAC8* | *YEL013W* | Phosphorylated and palmitoylated vacuolar membrane protein interacts with Atg13p, required for cytoplasm-to-vacuole targeting (Cvt) pathway; interacts with Nvj1p to form nucleus-vacuole junctions | | | | | | | |
| 1.8 | *VAM3* | *YOR106W* | Syntaxin-related protein required for vacuolar assembly; functions with Vam7p in vacuolar protein trafficking; multi specificity vacuolar t-SNARE mediating docking/fusion of multiple distinct late transport intermediates with vacuole | | | | | | | |
| 1.7 | *VPS41* | *YDR080W* | Vacuolar membrane protein, subunit of homotypic vacuole fusion and vacuole protein sorting (HOPS) complex; essential for membrane docking and fusion at Golgi-to-endosome and endosome-to-vacuole stages of protein transport | | | | | | | |
| 1.5 | *XRS2* | *YDR369C* | Protein required for DNA repair; component of Mre11 complex, involved in double strand breaks, meiotic recombination, telomere maintenance, and checkpoint signaling | | | | | | | |
| *0010526* | | *negative regulation of transposition, RNA-mediated* | *0.0063* | *0.0667* | | | *1.25E-02* | *K* | | *RTT103,PAF1,SIN3,BUD27,ASC1,PBS2* |
| 1.3 | *BUD27* | *YFL023W* | Unconventional prefoldin protein involved in translation initiation; mutants have inappropriate expression of nutrient sensitive genes due to translational derepression of Gcn4p transcription factor | | | | | | | |
| 1.3 | *PAF1* | *YBR279W* | Component of Paf1p complex, binds to and modulates RNA polymerases I and II activity; required for expression of a cell cycle-regulated genes | | | | | | | |
| 1.3 | *RTT103* | *YDR289C* | Protein interacts with exonuclease Rat1p and Rai1p; role in transcription termination by RNA polymerase II; regulation of Ty1 transposition | | | | | | | |
| 3.2 | *SIN3* | *YOL004W* | Component of Sin3p-Rpd3p histone deacetylase complex, involved in transcriptional repression and activation of diverse processes, including mating-type switching and meiosis; maintenance of chromosomal integrity | | | | | | | |
| *0055086* | | *nucleobase, nucleoside and nucleotide metabolic process* | *0.0338* | *0.1778* | | | *2.54E-05* | *T* | | *TFP3,VMA5,GPA2,TKL1,VMA2,NPT1,VMA13,VMA4,*  *VMA7,VPH1,ADO1,PPA1,CUP5,TFP1,VMA6,VMA8* |
| 1.8 | *ADO1* | *YJR105W* | Adenosine kinase, required for utilization of S-adenosylmethionine; involved in recycling adenosine produced through methyl cycle | | | | | | | |
| 1.2 | *TKL1* | *YPR074C* | Transketolase; catalyzes conversion of xylulose-5-phosphate and ribose-5-phosphate to sedoheptulose-7-phosphate and glyceraldehyde-3-phosphate in the pentose phosphate pathway; needed for synthesis of aromatic amino acids; TKL1 has a paralog, TKL2, that arose from the whole genome duplication | | | | | | | |
| 5.6 | *NPT1* | *YOR209C* | Nicotinate phosphoribosyltransferase, salvage pathway of NAD^+^ biosynthesis; required for silencing at rDNA, telomeres and mating-type loci | | | | | | | |
|  | *0007154* | *cell communication* | *0.059* | *0.1556* | | | *3.78E-01* | *K* | | *GPA2,PLC1,SLM4,GPR1,ATG7,MKS1,PEX1,HOG1,*  *ASC1,OPI1,TCO89,SNX4,CCZ1,PBS2* |
| 1.6 | *MKS1* | *YNL076W* | Pleiotropic negative transcriptional regulator involved in Ras-CAMP and lysine biosynthetic pathways and nitrogen regulation; involved in retrograde (RTG) mitochondria-to-nucleus signaling | | | | | | | |
| 1.7 | *OPI1* | *YHL020C* | Transcriptional regulator of a variety of genes; phosphorylation by protein kinase A stimulates Opi1p function in negative regulation of phospholipid biosynthetic genes; involved in telomere maintenance | | | | | | | |
| 1.2 | *PEX1* | *YKL197C* | AAA-peroxin heterodimerizes with AAA-peroxin Pex6p; recycling of peroxisomal signal receptor Pex5p from peroxisomal membrane to cystosol; induced by oleic acid and upregulated during anaerobiosis | | | | | | | |
| 4.2 | *PLC1* | *YPL268W* | Phospholipase C, generation of signaling molecules inositol 1,4,5-triphosphate (IP3) and 1,2-diacylglycerol (DAG); involved in regulating many cellular processes | | | | | | | |
| *0031326* | | *regulation of cellular biosynthetic process* | *0.1691* | *0.3222* | | | *1.14E-01* | *K* | | *TFP3,VMA5,GPA2,RTT103,ZAP1,HFI1,PAF1,SNF5,*  *VMA2,SAP30,DST1,TOM1,ZUO1,MKS1,SIN3,NPT1,IES6,HOG1,VPH1,ASC1,OPI1,TFP1,RXT3,DOA4,VMA6,PAT1,CCR4,SPT4,VMA8* |
| *0010468* | | *regulation of gene expression* | *0.1661* | *0.3* | | | *4.56E-01* | *K* | | *TFP3,VMA5,RTT103,ZAP1,HFI1,PAF1,SNF5,VMA2,*  *SAP30,DST1,TOM1,ZUO1,MKS1,SIN3,NPT1,IES6,HOG1,VPH1,ASC1,OPI1,TFP1,RXT3,VMA6,PAT1,CCR4,SPT4,*  *VMA8* |
| *0006351* | | *transcription, DNA-dependent* | *0.1204* | *0.2111* | | | *1* | *T* | | *RTT103,ZAP1,HFI1,PAF1,MEH1,SNF5,SAP30,DST1,*  *TOM1,MKS1,SIN3,NPT1,IES6,VMA10,HOG1,OPI1,RXT3,CCR4,SPT4* |
| *0070210* | | *Rpd3L-Expanded complex* | *0.003* | *0.0333* | | | *0.410236* | *K* | | *SAP30,SIN3,RXT3* |
| 1.5 | *CCR4* | *YAL021C* | Component of the CCR4-NOT transcriptional complex, which is involved in regulation of gene expression; component of the major cytoplasmic deadenylase, which is involved in mRNA poly(A) tail shortening | | | | | | | |
| 1.2 | *DST1* | *YGL043W* | General transcription elongation factor TFIIS, enables RNA polymerase II to read through blocks to elongation by stimulating cleavage of nascent transcripts stalled at transcription arrest sites | | | | | | | |
| 2.3 | *HFI1* | *YPL254W* | Adaptor protein required for integrity of SAGA complex, a histone acetyltransferase-coactivator complex involved in global regulation of gene expression through acetylation and transcription functions | | | | | | | |
| 1.2 | *IES6* | *YEL044W* | Protein associates with INO80 chromatin remodeling complex under low-salt conditions | | | | | | | |
| 1.6 | *PAT1* | *YCR077C* | rDNA locus stability, and protection of mRNA 3'-UTRs from trimming; functionally linked to Pab1p | | | | | | | |
| 1.6 | *SAP30* | *YMR263W* | Subunit of a histone deacetylase complex, along with Rpd3p and Sin3p, involved in silencing at telomeres, rDNA, and silent mating-type loci; involved in telomere maintenance | | | | | | | |
| 1.7 | *SPT4* | *YGR063C* | regulates Pol I and Pol II transcription, pre-mRNA processing, kinetochore function, and gene silencing; forms a complex with Spt5p | | | | | | | |
| 2.6 | *SNF5* | *YBR289W* | Subunit of SWI/SNF chromatin remodeling complex involved in transcriptional regulation; functions interdependently in transcriptional activation with Snf2p and Snf6p | | | | | | | |
| 1.5 | *TOM1* | *YDR457W* | E3 ubiquitin ligase of hect-domain class; role in mRNA export from nucleus, regulates transcriptional coactivators; involved in histone degradation | | | | | | | |
| 1.5 | *ZUO1* | *YGR285C* | Cytosolic ribosome-associated chaperone acts, with Ssz1p and Ssb proteins, as chaperone for nascent polypeptide chains | | | | | | | |
| *0006950* | | *response to stress* | *0.1305* | *0.1667* | | | *1* | *F* | | *DCC1,SNF5,ATG7,SSQ1,SIN3,PUG1,HOG1,TPS1,*  *OPI1,TPS2,TCO89,CCZ1,PBS2,HXK1,XRS2* |
| 1.4 | *HXK1* | *YFR053C* | Hexokinase isoenzyme 1 catalyzes glucose phosphorylation during glucose metabolism; highest expression during growth on non-glucose carbon sources; glucose-induced repression involves hexokinase Hxk2p | | | | | | | |
| 1.5 | *PUG1* | *YER185W* | Plasma membrane protein; uptake of protoprophyrin IX and efflux of heme; expression induced under low-heme and low-oxygen conditions; member of fungal lipid-translocating exporter (LTE) family | | | | | | | |
| 1.9 | *TPS1* | *YBR126C* | Synthase subunit of trehalose-6-phosphate synthase/phosphatase complex, synthesizes storage carbohydrate trehalose; expression induced by stress response and repressed byRas-cAMP pathway | | | | | | | |
| 6.0 | *TPS2* | *YDR074W* | Phosphatase subunit of trehalose-6-phosphate synthase/phosphatase complex, synthesizes storage carbohydrate trehalose; expression induced by stress conditions and repressed by Ras-cAMP pathway | | | | | | | |
| *0016043* | | *cellular component organization* | *0.2824* | *0.5222* | | | *5.89E-04* | *K* | | *VMA22,RTT103,VAM3,DBF2,HFI1,SLM4,PAF1,DCC1,*  *MEH1,SNF5,NUP188,MSW1,ATG7,SAP30,RBL2,DST1,*  *VPS41,TOM1,PEX1,SIN3,PKR1,VMA10,BUD27,VMA4,*  *VPH1,OCT1,NUP133,OPI1,CNM67,TCO89,SNX4,CUP5,TPM1,RXT3,DOA4,SPC72,PAT1,BUD31,CIS1,RAV1,*  *RAV2,CCZ1,PBS2,VAC8,SPT4,VPH2,XRS2* |
| *0006996* | | *organelle organization* | *0.1824* | *0.3333* | | | *1.77E-01* | *T* | | *VAM3,DBF2,HFI1,PAF1,DCC1,SNF5,NUP188,MSW1,*  *SAP30,RBL2,VPS41,TOM1,PEX1,SIN3,VMA10,VMA4,*  *OCT1,NUP133,OPI1,CNM67,CUP5,TPM1,RXT3,SPC72,CIS1,CCZ1,PBS2,VAC8,SPT4,XRS2* |
| 2.3 | *BUD31* | *YCR063W* | Component of the SF3b subcomplex of the U2 snRNP; diploid mutants display a random budding pattern instead of the wild-type bipolar pattern; facilitates passage through G1/S Start, but is not required for G2/M transition or exit from mitosis | | | | | | | |
| 1.2 | *CNM67* | *YNL225C* | Component of spindle pole body outer plaque; required for spindle orientation and mitotic nuclear migration | | | | | | | |
| 1.5 | *MSW1* | *YDR268W* | Mitochondrial tryptophanyl-tRNA synthetase | | | | | | | |
| 1.2 | *NUP133* | *YKR082W* | Subunit of Nup84p subcomplex of nuclear pore complex (NPC), localizes to both sides of NPC, required to establish a normal nucleocytoplasmic concentration gradient of GTPase Gsp1p | | | | | | | |
| 1.5 | *NUP188* | *YML103C* | Subunit of nuclear pore complex (NPC), involved in structural organization of complex and nuclear envelope, involved in nuclear envelope permeability, interacts with Pom152p and Nic96p | | | | | | | |
| 2.0 | *RBL2* | *YOR265W* | Protein required for microtubule morphogenesis, protection from excess free beta-tubulin; proposed to be involved the folding of beta-tubulin | | | | | | | |
| 1.7 | *SPC72* | *YAL047C* | Component of cytoplasmic Tub4p (gamma-tubulin) complex, links spindle pole bodies to microtubules; astral microtubule formation and stabilization | | | | | | | |
| 1.2 | *TPM1* | *YNL079C* | Major tropomyosin isoform; stabilizes actin cables and filaments to direct polarized cell growth and distribution of several organelles | | | | | | | |
| *0034613* | | *cellular protein localization* | *0.0557* | *0.1556* | | | *2.17E-01* | *T* | | *VAM3,PLC1,NUP188,ATG7,VPS41,PEX1,GSF2,OCT1,*  *NUP133,SNX4,CUP5,CCZ1,PBS2,VAC8* |
| 1.4 | *GSF2* | *YML048W* | ER localized integral membrane protein, may promote secretion of certain hexose transporters, including Gal2p; involved in glucose-dependent repression | | | | | | | |
| *0016070* | | *RNA metabolic process* | *0.2507* | *0.3* | | | *1* | *T* | | *RTT103,LSM1,VAM3,ZAP1,HFI1,PAF1,MEH1,SNF5,*  *MSW1,SNT309,SAP30,DST1,TOM1,MKS1,SIN3,NPT1,*  *IES6,VMA10,BUD27,HOG1,TRK1,OPI1,RXT3,PAT1,*  *BUD31,CCR4,SPT4* |
| *0000375* | | *RNA splicing, via trans esterification reactions* | *0.0179* | *0.0333* | | | *1* | *K* | | *LSM1 SNT309 BUD31* |
| 1.3 | *LSM1* | *YJL124C* | Lsm (Like Sm) protein; forms heteroheptameric complex (with Lsm2p, Lsm3p, Lsm4p, Lsm5p, Lsm6p, and Lsm7p) involved in degradation of cytoplasmic mRNAs; forms cytoplasmic foci upon DNA replication stress | | | | | | | |
| 1.3 | *SNT309* | *YPR101W* | Member of the NineTeen Complex (NTC) that contains Prp19p and stabilizes U6 snRNA in catalytic forms of the spliceosome containing U2, U5, and U6 snRNAs; interacts physically and genetically with Prp19p | | | | | | | |
| *0000910* | | *cytokinesis* | *0.0181* | *0.0333* | | | *1* | *K* | | *BUD27,BUD31,CYK3* |
| 1.5 | *CYK3* | *YDL117W* | SH3-domain protein located in mother-bud neck and cytokinetic actin ring; suggested role in cytokinesis | | | | | | | |
|  | *0001403* | *invasive growth in response to glucose limitation* | *0.0077* | *0.0333* | | | *1* | *K* | | *GPR1,DIA2,ASC1* |
| 1.2 | DIA2 | YOR080W | Origin-binding F-box protein; forms SCF ubiquitin ligase complex with Skp1p and Cdc53p; role in DNA replication, transcription; assembly of RSC complex and RSC-mediated transcription regulation | | | | | | | |
| *0006629* | | *lipid metabolic process* | *0.0453* | *0.0556* | | | *1* | *F* | | *PLC1,MCT1,RML2,ERG6,OPI1* |
| *0006066* | | *cellular alcohol metabolic process* | *0.0359* | *0.0556* | | | *1* | *F* | | *TKL1,PLC1,ERG6,TCO89,HXK1* |
| 1.9 | *ERG6* | *YML008C* | Delta(24)-sterol C-methyltransferase, converts zymosterol to fecosterol in ergosterol biosynthetic pathway; localized to lipid particles and mitochondrial outer membrane | | | | | | | |
| 1.5 | *RML2* | *YEL050C* | Mitochondrial ribosomal protein of large subunit; fat21 mutant allele causes inability to utilize oleate and may interfere with activity of the Adr1p transcription factor | | | | | | | |
| 1.2 | *MCT1* | *YOR221C* | Predicted malonyl-CoA:ACP transferase, putative component of a type-II mitochondrial fatty acid synthase; phospholipid remodeling | | | | | | | |
| *0006974* | | *response to DNA damage stimulus* | *Slim mapper* | | | | *PAF1,SNF5,DCC1,XRS2,YDR433W,SPT4,NUP133,SIN3* | | | |
| 1.5 | *YDR433W* | *YDR433W* | Dubious open reading frame unlikely to encode a functional protein | | | | | | | |
| *other* | | *other* | *Slim mapper* | | | *PUG1, YGR064W* | | | | |
| 1.5 | *YGR064W* | *YGR064W* | Dubious open reading frame unlikely to encode a protein | | | | | | | |
| *0008150* | | *biological process unknown* | *Slim mapper* | | *YCL007C,IES6,YFL012W,YLL007C,DUF1* | | | | | |
| 1.7 | *YCL007C* | *YCL007C* | Dubious open reading frame unlikely to encode a protein | | | | | | | |
| 1.7 | *YLL007C* | *YLL007C* | Putative protein of unknown function | | | | | | | |
| 1.3 | *YFL012W* | *YFL012W* | Putative protein of unknown function; transcribed during sporulation | | | | | | | |
| 1.4 | *DUF1* | *YOL087C* | Putative protein of unknown function | | | | | | | |
